# Formalization of the classification pattern: survey of classification modeling in information systems engineering

**DOI:** 10.1007/s10270-016-0521-5

**Published:** 2016-04-16

**Authors:** Chris Partridge, Sergio de Cesare, Andrew Mitchell, James Odell

**Affiliations:** 1 Brunel University London, London, UK; 2 BORO Solutions, London, UK; 3 James Odell Associates, Ann Arbor, MI USA

**Keywords:** Classification system, Classification, Powerset, Powertype, Set theory

## Abstract

Formalization is becoming more common in all stages of the development of information systems, as a better understanding of its benefits emerges. Classification systems are ubiquitous, no more so than in domain modeling. The classification pattern that underlies these systems provides a good case study of the move toward formalization in part because it illustrates some of the barriers to formalization, including the formal complexity of the pattern and the ontological issues surrounding the “one and the many.” Powersets are a way of characterizing the (complex) formal structure of the classification pattern, and their formalization has been extensively studied in mathematics since Cantor’s work in the late nineteenth century. One can use this formalization to develop a useful benchmark. There are various communities within information systems engineering (ISE) that are gradually working toward a formalization of the classification pattern. However, for most of these communities, this work is incomplete, in that they have not yet arrived at a solution with the expressiveness of the powerset benchmark. This contrasts with the early smooth adoption of powerset by other information systems communities to, for example, formalize relations. One way of understanding the varying rates of adoption is recognizing that the different communities have different historical baggage. Many conceptual modeling communities emerged from work done on database design, and this creates hurdles to the adoption of the high level of expressiveness of powersets. Another relevant factor is that these communities also often feel, particularly in the case of domain modeling, a responsibility to explain the semantics of whatever formal structures they adopt. This paper aims to make sense of the formalization of the classification pattern in ISE and surveys its history through the literature, starting from the relevant theoretical works of the mathematical literature and gradually shifting focus to the ISE literature. The literature survey follows the evolution of ISE’s understanding of how to formalize the classification pattern. The various proposals are assessed using the classical example of classification; the Linnaean taxonomy formalized using powersets as a benchmark for formal expressiveness. The broad conclusion of the survey is that (1) the ISE community is currently in the early stages of the process of understanding how to formalize the classification pattern, particularly in the requirements for expressiveness exemplified by powersets, and (2) that there is an opportunity to intervene and speed up the process of adoption by clarifying this expressiveness. Given the central place that the classification pattern has in domain modeling, this intervention has the potential to lead to significant improvements.

## Introduction

Classification (in the everyday sense) is ubiquitous [28, 36, 102]. This should not be surprising; classifications are one of the major ways we organize things in the world. Biologists classify our species as *Homo sapiens* and our pet dogs as *Canis lupus familiaris*; governments classify us in all sorts of ways. Wherever we need to organize information of any size, indeed whenever we think, we start classifying. In “Primitive Classification,” Durkheim and Mauss [28] argued that the sophistication of the classification system reflected the sophistication of the culture using it—more sophisticated cultures used more sophisticated classifications. In “The Order of Things,” Foucault [36] described how classification systems have evolved over time.

The history of information shows an obvious correlation between the amount of information available and the nature of its classification; as the amount of information grows, more instances of classification as well as more sophisticated classification structures emerge (Ong [86]). Furthermore, the nature of the storage medium plays a role in shaping the kind of structures that emerge (Olson [82]).

One would expect the emergence of computer systems to follow this evolutionary pattern. It can already be seen that computers lead to substantial increases in the amount of information and how this has led to a need for better classification systems. Computers, unlike their paper precursors, are formal systems; this suggests that one avenue for improvement would be a more formal structure.

However, there is currently no survey that examines this. This paper aims to start to fill that gap. It surveys how the formalization of the classification pattern has emerged and evolved in information systems engineering (ISE). It briefly tracks the history of its emergence and builds a picture of the current status.

This survey identifies a small number of communities currently working in this area, with different approaches and with different underlying formalizations. This necessitates the creation of a framework and benchmark against which to assess them. A classic example, the Linnaean classification, is chosen as a benchmark example. A mathematical framework is developed for characterizing the formal structure of classification, and this is used to expose the formal structure of the chosen example. This is described in the first part of the paper.

With this framework and benchmark in place, the ISE literature is reviewed revealing a variety of emerging approaches. Their underlying formalizations are analyzed, benchmarked, and compared. This exposes a general slow adoption over time of the formal structures needed for the classification pattern, as well as different adoption routes and stages in different communities. The conclusion of this research is that the way to formalize classification is being explored by the ISE community, but that this has not yet arrived at a mature stable mainstream state. The hypothesis is that both the formal complexity of the pattern and the requirement for an explanation of what the formal structures being proposed represent (a semantic-ontological narrative about what aspect of reality they are reflecting) contribute to the slow adoption. This is described in the second part of the paper.

### Why classification is being formalized now

As a community acquires more information, this creates a corresponding need to improve its classification systems. For example, if a community is originally only interested in five or ten objects in a domain, there is little need for a sophisticated classification system. When this increases to a couple of hundred, there is need for a simple system. For example, Lakoff’s [66] title “Women, Fire and Dangerous Things” refers to one of the four basic categories hard-wired into the language of a preliterate community. When this increases to thousands or millions, the need for a more sophisticated system becomes overwhelming.

Observers of information technology revolutions (such as Ong [86] and Olson [82]) have noted that these tend to encourage developments in classification systems, driven in part by increases in the volume of information. They give as examples one of the earliest systems of classification, the Aristotelian categories, which emerged in Ancient Greece as writing was establishing itself, and the Linnaean classification, which emerged as printing established itself.

There seems to be a similar situation now with the computing (information technology) revolution. Many of the methods of classification used currently were developed for paper technology, prior to the emergence of computing; however, the classifications and the data they classify are now typically stored on computers. Computer storage not only offers opportunities for increasing the amount of data stored, it also offers opportunities for structuring the data in ways that paper technology does not. Computers operate within more formal structures, so one of the first hurdles facing the more informal, implicit paper-based classification patterns in their migration to computer systems is formalization. This then opens the door to opportunities for innovation and improvement, to deal with the increases in the volume of data. This paper starts to look within ISE at how the formalization of the techniques for classifying has emerged and developed.

### What is the classification pattern?

The term “classification” has a variety of senses, typically associated with kinds of classification systems. Our interest is in a pattern that can be discerned at the core of these classification systems, which underpins its structure, what we therefore call the “classification pattern.” In our formal analysis, we highlight this pattern and characterize its structure. Our focus on this specific pattern is to enable us to make clearer comparisons. It is not to dismiss any of the other senses.

### Separating the concerns

One of the issues faced when starting to build up a picture of the classification pattern in computing is that there were several competing concerns. As well as the requirement to represent the classifications in the domain, there are competing implementation requirements on the representation that distort the picture.

To resolve this, the question of what is being represented is separated from how it is implemented in a particular system. The survey is only concerned with the first aspect—what is being represented—independent of any implementation requirement.

This kind of separation of concerns approach is well established in computing and software engineering. The Object Management Group’s (OMG) Model-Driven Architecture (MDA) is a well-known example; in their terms, the focus here is on the Computational Independent Model (CIM).

It is also important to separate the core of the classification pattern, whose formal structure can be exemplified by powersets and subsets, from more general peripheral concerns. For example, powersets are a mechanism for generating higher-order sets (types). However, as the formal exposition below shows, higher-order sets (types) are not sufficient, by themselves, to characterize the classification pattern. Indeed, from some perspectives, they are not even necessary, as illustrated by the communities who have developed classification pattern characterizations that deliberately exclude them (as described below). While higher-order types can usefully be characterized using a particular notion of powertypes as powersets, this dependence is not symmetrical. Hence, while higher-order types may be a natural extension of one approach to the classification pattern, they are not a core element of it and so not a core concern of this paper. They are nonetheless important, and so we discuss future research into them in Sect. 8.

### How to assess the formalization

There are a number of communities currently working in this area, with different approaches and different underlying formalizations. These are, broadly speaking, communities with an interest in conceptual modeling. To assess their approaches, a benchmark framework was developed as described in the first part of the paper. The benchmark framework has three components.

The first is an example of classification that is sufficiently rich to illustrate a reasonably broad set of requirements. For this, a classic example of classification from biology is used, the Linnaean classification. This is often seen in the literature on classification and because of its richness can be regarded as a classification system.

The second is a formal structure for classification. We use a mathematical theory, set theory, with a particular emphasis on the mathematical object, the powerset (often called powertype in computing) and associated objects. The purpose of this structure was to provide sufficient formal detail to benchmark the classification structures under analysis. It is not intended to be a fully fledged formalization, to, for example, stand shoulder to shoulder and compete with the approaches reviewed here. Nor is there intended to be any suggestion that set theory (and its semantics) is the only possible way of formalizing these structures (indeed, as discussed below, there are competing formal approaches within mathematics). For us, it is one useful way of characterizing the structure we are looking at so we can benchmark it.

The third component is built from the preceding components, it is an analysis of the selected example’s formal structure using the mathematical objects. This gives an insight into the underlying formal structure of the classification pattern.

### ISE survey: assessing the formalization

The benchmark framework was used to survey the evolution of the formalization. The survey starts by taking a brief general look at the adoption of the mathematical structures, to provide a benchmark against which to measure their adoption of a formalized classification structure.

The paper then surveys the formalization by community. Three communities were found making significant contributions in this area; these communities are reviewed in some detail. The completeness of their formalization was analyzed against the formalized benchmark. Moreover, the literature of a number of communities making indirect contributions was reviewed. This is described in the second part of the paper. Finally, a summary of the survey is provided, looking at how far the adoption has progressed across the communities, both from the perspective of the mathematical structures and from the benchmark requirements.

**Fig. 1:**
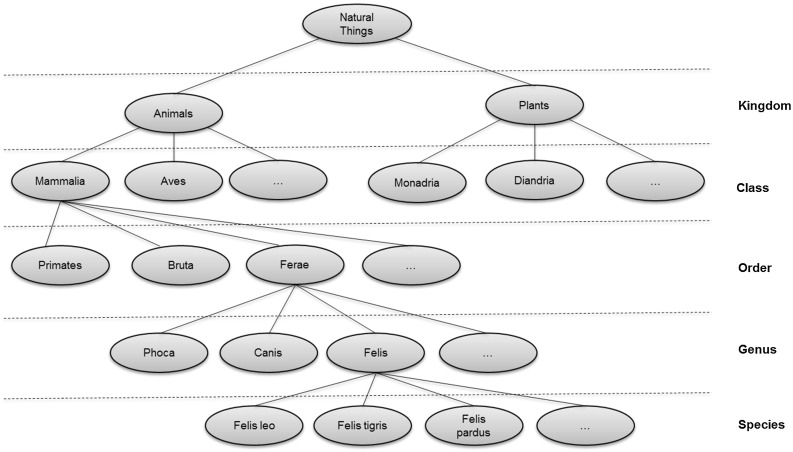
Linnaean classification scheme

**Fig. 2:**
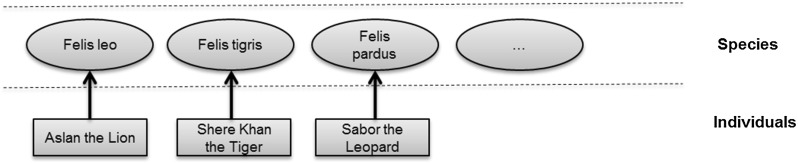
Example individuals

## The classification benchmark

In this section, the classic example, i.e., the Linnaean biological classification, is described. This example was chosen as the benchmark for the surveyed literature. The example is reasonably sophisticated and helps to illustrate the level of complexity that appears in real situations. An important benefit of choosing a classical biological example is that it is well studied. This does not mean the example is not relevant in other areas; there are many examples in business with a similar kind of classification structure; ISO 10962—Classification of Financial Instruments is a modern example.

Biological classification is one of the earliest modern (after the emergence of printing) systems of classification. It is crystallized in the ranked system of Carl Linnaeus upon which the current Nomenclature Codes are based. One standard definition of the classification is Mayr and Bock [75]: “The arrangement of entities in a hierarchical series of nested classes, in which similar or related classes at one hierarchical level are combined comprehensively into more inclusive classes at the next higher level.”

**Fig. 3:**
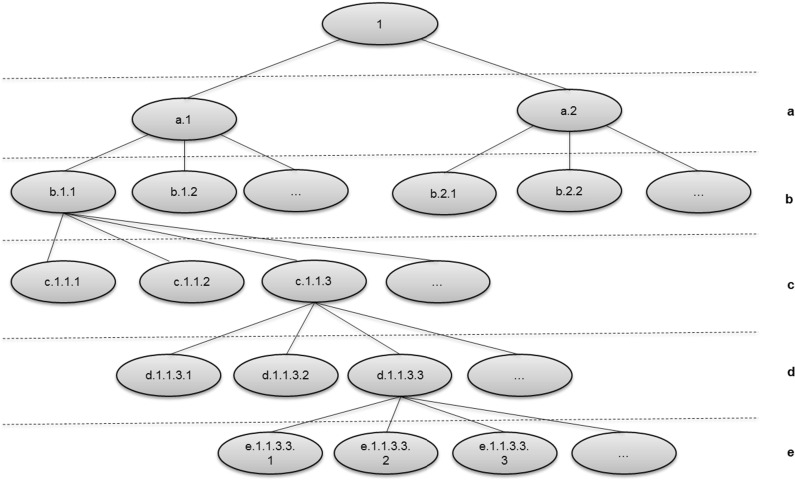
Structure of the Linnaean classification scheme

Carl Linnaeus published his classification system in the book Systema Naturae. This went through several editions, the first being published in 1735. The Linnaean system, in its original form, represented a classification of all natural things (including animals, plants, and minerals). In its modern equivalent, it is primarily used as a classification of living organisms (animals and plants).

The Linnaean system classifies livings organisms at different levels known as ranks. In this example, there are five ranks: Kingdom, Class, Order, Genus, and Species. Each rank breaks down the classifications of the previous rank into finer detail. Figure 1 illustrates this breakdown. Individual animals are typically shown as instances of the lowest level rank, Species; Fig. 2 has some examples. (Class is an overloaded term, but it should be clear from the context here that “Class” means Linnaean class and not some other sense; for example object-oriented class or set-theoretic class).

Linnaeus’s Systema Naturae went through several editions, with the classification updated in each. Subsequently, the classification continued to evolve. One aspect of the evolution was the emergence of different bases for the classification, for example, morphology-based phenetics and ancestor-based cladistics. More recently, Ghiselin [39] and Hull [54] suggest that instead of viewing species as natural kinds, they should be thought of as individuals. For the benchmark example, the question of the “correct” classification is not material. What is required is merely an example of a sufficiently sophisticated classification structure. Hence, a basic Linnaean structure is adopted, as this is adequate for almost all the needs of this paper.

There is an aspect of classification that this simple Linnaean example by its nature does not illustrate; this is that something can be classified in multiple ways. Within the example, each individual organism is classified once and only once—at the lowest rank. To see that this might not be the whole picture, consider the phenetic and cladistic classifications mentioned above. In a system with both classifications, some individual organisms will be classified twice, once by each of the two systems, and some classifications will have multiple parents. This is not an odd extreme situation. There are classification systems with this kind of multiple classification built into their framework. The colon classification developed by Ranganathan [101] for libraries is an unambiguous example. This has multiple classification taxonomies called facets, and every document is multiply classified under each facet. This is sufficient evidence that multiple classification is a requirement that should be supported by a reasonably sophisticated classification pattern.

### Formal structure

This paper is concerned with the formal structure of the chosen classification scheme. One common way of illustrating this is by substituting meaningless labels for names, showing the structure without the content (for a classic example, see the railroad map in Carnap’s The Logical Structure of the World [16]). This is done for Figs. 1 in 3. The aim of this work was to characterize the nature of this structure, irrespective of the content; elements of which would re-appear in other classifications.

## Mathematical background

Modern mathematics can be seen as the science of formal patterns, as described by Devlin [27] and Shapiro [105]. This makes it a good tool for capturing the formal structure of the classification pattern, such as that in Fig. 3. This section describes the mathematical objects needed for this.

### Which mathematical theory?

There is a choice of theories from which to select the required objects. The foundations of mathematics are an active research area, and there are three broad mathematical theories in play. In historical order of emergence, these are set theory, type theory, and category theory. All these theories contain the resources to characterize the classification pattern and much else. While there are technical differences between the theories, these differences are not relevant for the purposes of this paper. In principle, the framework could be based upon any (or all) of the three theories. However, to simplify the exposition, this paper is based on one theory, set theory, as it is the most approachable for the non-specialist. For the interested reader, a brief overview of such differences and the relations between the theories is provided at the end of this section along with some useful references.

### Scope

Only a small core of the theory is required for the formalization of the classification pattern. The interest of this study centers primarily on the mathematical object that set theory calls “powerset” and its associated objects, such as “set” and “subset.” Analogous objects appear in all three theories, sometimes with different suffixes. In type theory, the suffix “type” is used instead of set; it has types, powertypes, and subtypes. In category theory, there are objects called “set,” “powerset,” and “subset,” and these have been generalized to “objects,” “powerobjects,” and “subobjects.”

In the literature, these terms are sometimes spelled as a single word (“powerset,” “powertype,” etc.), while at other times the two-word form is used (“power set,” “power type,” etc.). In this paper, the single word form will be adopted.

This section will focus on the powerset, core to the formal structure of classification, and provide a brief overview of the formal structure of powersets and associated mathematical objects. Powersets are introduced in the next section from a simple historical perspective; for more detail on the early history, see Ferreirós [31], Kanamori [61], Van Heijenoort [117], and Grattan-Guinness [44].

### Powersets and related mathematical objects

As subsequent sections will show, the ISE literature surveyed normally does not always have a sufficiently clear understanding of the mathematical objects underpinning the mathematical framework that this paper adopts. Hence, care is taken to describe the mathematical underpinnings in this section. Readers familiar with set theory can skim or skip this section. To assist the reader, the key symbols used are explained in Appendix.

#### Origin and definition of powerset

Set theory is a core part of modern mathematics and is often employed as a foundational system for the whole of the discipline. Powerset is a key part of the theory and is commonplace in mathematics. Its origin can be traced back to Cantor’s [12] diagonalization theorem, which used but did not explicitly mention powerset. The first explicit mention of powerset is in Zermelo’s [120] axiomatization of set theory, which includes among its axioms AXIOM IV: Axiom of the powerset (Axiom der Potenzmenge):

1 $$\begin{aligned} \forall {x}\,\exists {y}\,\forall {z}\left[ { {z}\in {y}\equiv \forall {w}\left( { {w}\in {z}\rightarrow {w}\in {x}} \right) } \right] \end{aligned}$$

Or, informally:

To every set T, there corresponds a set T’, the powerset of T, that contains as elements precisely all subsets of T.

It is from Zermelo’s axiomatization that powersets then became commonplace in mathematics. Zermelo’s axiomatization was developed by Abraham Fraenkel retaining the powerset axiom, and the resultant Zermelo–Fraenkel theory, known as ZF, is the basis for the standard axiomatization used in mathematics today. In modern mathematics, the powerset of A is usually written as $\wp (\hbox {A})$ (where $\wp$ is called the “Weierstrass p”). This convention shall be followed here.

A simple example will help to illustrate what a powerset is. Consider the following set:

$$\begin{aligned} A\equiv \left\{ {\hbox {Africa},\hbox {Asia},\hbox {Europe}} \right\} \end{aligned}$$

The powerset of A or $\wp ({\hbox {A}})$ is a set that has as members all the subsets of A; therefore,

$$\begin{aligned} \wp (A)\equiv & {} \left\{ \,\,\left\{ {\hbox {Africa}} \right\} ,\,\left\{ {\hbox {Asia}} \right\} ,\,\left\{ {\hbox {Europe}} \right\} ,\,\left\{ {\hbox {Africa},\,\hbox {Asia}} \right\} ,\right. \\&\left. \left\{ {\hbox {Asia},\,\hbox {Europe}} \right\} ,\,\left\{ {\hbox {Africa},\,\hbox {Europe}} \right\} ,\right. \\&\left. \left\{ {\hbox {Africa},\,\hbox {Asia},\,\hbox {Europe}} \right\} \,\right\} \end{aligned}$$

**Fig. 4:**
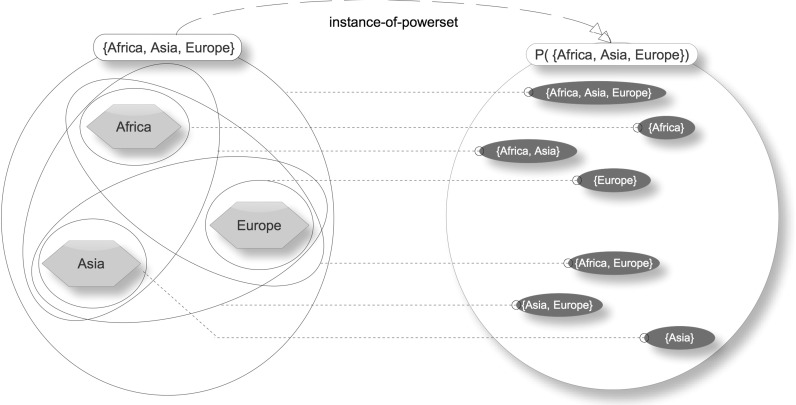
Example powerset

Figure 4 illustrates this example, showing visually that all subsets of A are members of $\wp ({\hbox {A}})$ and all members of $\wp ({\hbox {A}})$ are subsets of A. It also shows the instance-of-powerset relation between the power-instance and its power-set. Traditionally, the empty set is considered to be a member of every powerset; however, there are nonstandard approaches that eschew this. To simplify presentation, particularly in relation to the classification pattern, here and elsewhere in the paper, the empty set has deliberately been omitted from powersets.

The definition of a powerset uses the terms “set,” “members,” and “subsets.” These are part of a closely associated group of mathematical objects required in order to characterize the formal structure of classification. These elements are described in the following subsections.

#### Set (and members)

Sets are often described as collections of objects. There is some debate as to how close the natural notion of collections is to sets. For example, Black [10] suggests that there may be differences between set and collection, while Halmos [47] (p. 1) considers the two almost synonymous, stating:

- “A pack of wolves, a bunch of grapes, or a flock of pigeons are all examples of sets of things.” [47]

Although mathematicians worked with sets before Cantor, it is Cantor who is closely associated with them due to a few often-cited descriptions:

- “By a ‘set’ we understand any collection into a whole M of definite well distinguished objects m of our intuition or thought.” [13]
-  [A set as a] “many, which can be thought of as one, i.e., a totality of definite elements that can be combined into a whole by a law.” [13]

This “one over many” argument has roots going back to Plato; for example, in [100], he writes “We customarily hypothesize a single form in connection with each collection of many things to which we apply the same name.” Plato’s dialogues contain arguments against this position, for example the “third man argument” in [99]. This argument was taken up by Aristotle, and the debate has generated significant discussion; a recent example is Fine [33]. Cantor’s resolution, introducing an object that is both one and many, is now standard in set theory. However, a couple of the powertype strands we examine later do not accept the Cantorian resolution and propose a different approach. Hence, we use this formalization as a benchmark for classification functionality, rather than as a template for a solution.

There are little or no constraints on what a set can be. Sets are arbitrary, and any collection of objects in a domain qualifies as a set as described by Ferreirós [32]. One modern view is that sets are defined by the member-of relation. It is said that A is a member-of the set B (in symbols $\hbox {A} \in \hbox {B}$), or that the set B contains A as its element. The importance of the member-of relation is shown by the way the identity of a set is determined by its members; two sets are equal if they have exactly the same elements as members. In Zermelo’s [120] set theory, this was enshrined in AXIOM I: Axiom of extensionality (Axiom der Bestimmtheit):

2 $$\begin{aligned} \forall x\,\forall y [\forall z ( z \in x \equiv z\in y)\rightarrow x= y] \end{aligned}$$

Or, informally:

If every element of a set M is also an element of N and vice versa, then $\hbox {M} \equiv \hbox {N}$.

Briefly, every set is determined by its elements.

#### Ur-elements

Some objects in a domain do not have members, so they are not sets. These are traditionally known as ur-elements (from the German prefix ur-, “primordial”). In the simple example above (Fig. 4), Africa, Asia, and Europe are ur-elements. This distinction can be seen as having similar formal properties to the distinction between universals and particulars that started with Aristotle’s division into primary substance (particular, ur-element) and secondary substance (universal, set); in Categories, Aristotle [3] stated that primary substance cannot have instances, though it can be an instance, whereas a secondary substance typically has instances.

#### Subset-of

A subset is a set contained in another set. More formally, if A is a subset of B (this is equivalent to “B is a superset of A”) then every member-of A is also a member-of B. This can be written in a number of ways, as “*x* is a subset-of *y*” or “subset-of (*x*, *y*)” or “$x \subseteq y$” and is defined as:

3 $$\begin{aligned} x \subseteq y\,\hbox {iff}\,\,\forall z \,(z \in x \rightarrow z \in y). \end{aligned}$$

From this definition, it follows that a set is a subset-of itself, a property known as reflexivity.

4 $$\begin{aligned} \forall z (z\,\hbox {is a Set} \rightarrow z \subseteq z). \end{aligned}$$

From the definition, it also follows that the subset-of relation is transitive. A relation TR is transitive if *x*TR*y* (TR relates *x* to *y*) and *y*TR*z* implies that *x*TR*z*. Formally,

5 $$\begin{aligned} \forall x \,\forall y\,\forall z\,(x\hbox {TR}y \wedge y\hbox {TR}z) \rightarrow x\hbox {TR}z \end{aligned}$$

In terms of the subset-of relation, this schema becomes:

6 $$\begin{aligned} \forall a\,\forall b\,\forall c\,(a \subseteq b \wedge b \subseteq c) \rightarrow a \subseteq c \end{aligned}$$

Here is an example with the constants given a specific interpretation.

$$\begin{aligned} \hbox {A}\equiv & {} \hbox {(set of) animals}\\ \hbox {B}\equiv & {} \hbox {(set of) mammals}\\ \hbox {C}\equiv & {} \hbox {(set of) dogs} \end{aligned}$$

Here given that C is a subset-of B (i.e., all dogs are mammals) and B is a subset-of A (i.e., all mammals are animals), then C is a subset-of A (i.e., all dogs are animals). More formally,

7 $$\begin{aligned} (A \subseteq B \wedge B \subseteq C) \rightarrow A \subseteq C \end{aligned}$$

**Fig. 5:**
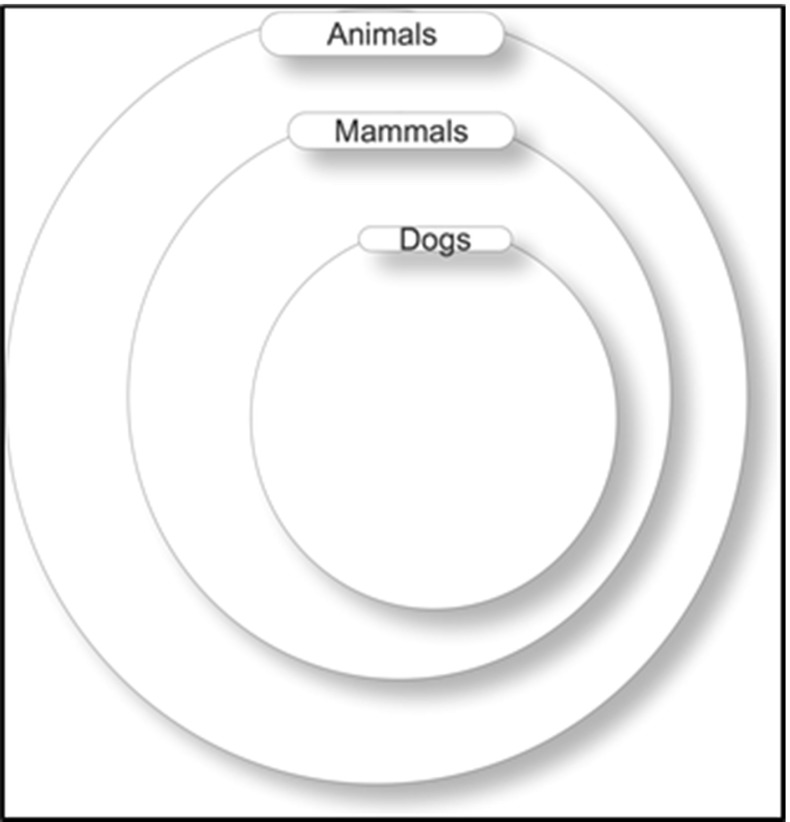
Subset transitivity

These relationships are illustrated in Fig. 5.

A common mistake for beginners is to conflate the subset-of and the member-of relations as, for example, noted in Partridge [88] and Kühne [63]. A good rule of thumb is that the subset relation is transitive, whereas the member-of relation is not. This member-of intransitivity stratifies the sets into a leveled hierarchy—in a way that the subset-of relation does not.

Subset-of (like sets) have few, if any, constraints. Given a set *X* and its members, then every combination of the members is also a set and a subset-of *X*. For example, given the set $X=\{1,2,3\}$, all of the following are sets with a subset-of relation to *X*:

1. {1, 2, 3},
2. {1, 2},
3. {2, 3},
4. {1},
5. {2},
6. {3}.

**Fig. 6:**
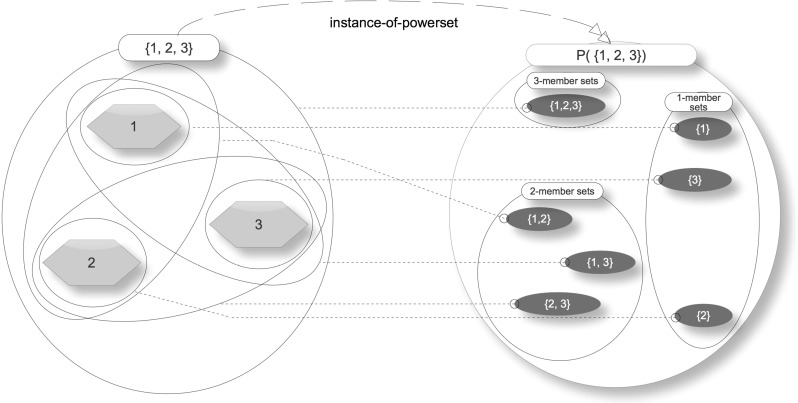
Example powerset

#### Powerset expanded

The objects defined above are required to understand the definition of powerset in Zermelo’s AXIOM IV (given above). The definition is expanded here with two of its consequences that show the relationship between subsets and members as this will prove useful in the exposition.

Given a set T and its powerset $\wp (\hbox {T})$:

1. All subsets of T are members of $\wp (\hbox {T})$.
2. All members of $\wp (\hbox {T})$ are subsets of T.

More formally,

8 $$\begin{aligned}&\forall T\,\forall z\,[(z \subseteq T) \rightarrow (z \in \wp (T))] \end{aligned}$$

9 $$\begin{aligned}&\forall T \,\forall z\,[(z \in \wp (T)) \rightarrow (z \subseteq T)] \end{aligned}$$

One way of viewing these two consequences is as closure rules. (8) can be seen as powerset-member closure—where any object that is recognized as a subset of the power-member (the set that is being “powerset-ed”) has also to be recognized as a member of the powerset. (9) correspondingly can be seen as a powerset-subset closure.

In standard set theory, each set has one and only one powerset, and vice versa, each powerset is a powerset of one and only one set. In modeling terms, this is usually stated as the powerset-of relation is one-to-one.

#### Set of subsets of a set (powerset-subset)

A non-empty collection of subsets of a given set S is called a set of subsets of S, or a set of sets over S or a family of subsets of S. This can be regarded as weaker than the powerset as it meets (9), but not necessarily (8); as all instances of the “set of subsets of S” are subsets of S, there may be subsets of S that are not instances of the “set of subsets of S.”

Another way of thinking of a “set of subsets of S” is of a subset of a powerset, a powerset-subset. The powerset of S will contain all the subsets of S. So any sets of subsets of S will be a subset of the powerset of S. The limiting case is where the set of subsets of S is all the subsets and so it is the powerset of S. The powerset of S is a powerset-subset of S because the subset relation is reflexive, so the powerset of S is a subset of itself.

Formalizing this Powerset-Subset-Of relation can be done by deconstructing it into already existing relations. If *x* is a powerset-subset of *y*, then *x* is a subset of the powerset of *y*. More formally,

10 $$\begin{aligned}&{\textit{Powerset-Subset-Of}} \equiv {\hbox {PSO}} \end{aligned}$$

11 $$\begin{aligned}&(<x,y>\in {\hbox {PSO}}) \equiv (x\subseteq \wp (y)) \end{aligned}$$

Powerset-subsets, as subsets of a powerset, are subject to the powerset-member closure mentioned above. In other words, every member of the powerset-subset is also a subset of power-member. However, it is not subject to powerset-subset closure, for obvious reasons.

It turns out that many simple classifications are powerset-subsets. This is illustrated in the following example. Consider the set {1, 2, 3}. Its powerset, $\wp (\{1,2,3\})$, is a set of all its subsets—shown in Fig. 6. As the figure shows, there are various subsets of the powerset (in other words, powerset-subsets) that classify the original set: three-member sets, two-member sets, and one-member sets. A more complicated classification system will take these powerset-subsets as a ranking of the classifications by number of members—a topic presented later in the paper when the Linnaean example is examined.

Unlike the powerset-of relation, the powerset-subset-of relation is many-to-many. Given a set of subsets of S, there are a number of other sets of which it could be the powertype-subset; any superset of S will be a candidate. Similarly, for a set S of a reasonable size, there will be a significant number of sets of subsets it could have. This makes the relation many-to-many. The following example will help to clarify this point.

**Fig. 7:**
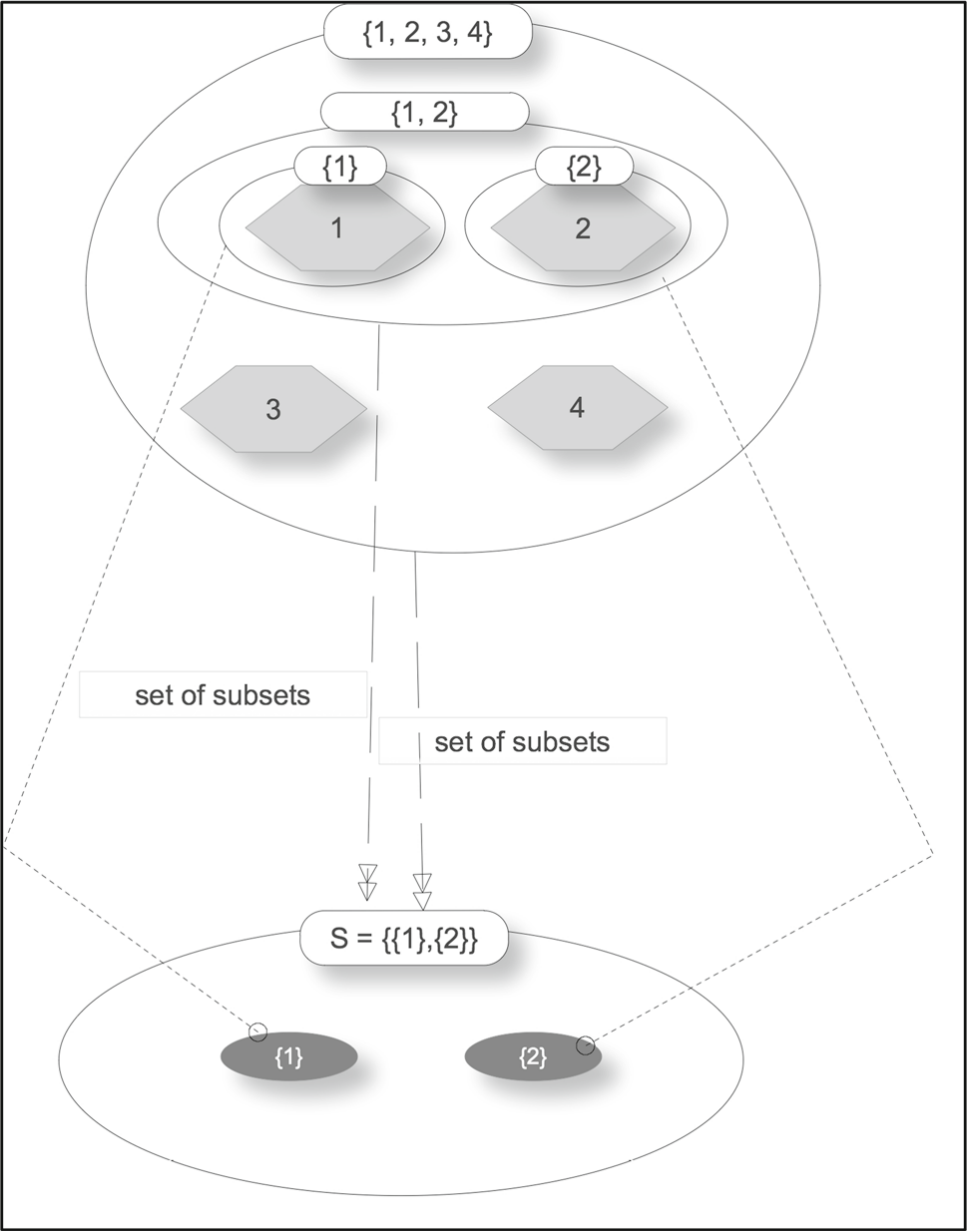
Many-to-many powerset-subset-of relation

Consider the set ${S}=\{\{1\},\{2\}\}$. S is a set of subsets for any set that has 1 or 2 as members, for example, any of the following sets and all their supersets qualify: {1, 2}, {1, 2, 3, 4}. Figure 7 represents this example.

#### Intersection and union of sets

Two important operations that can be conducted on sets are intersection and union. The intersection of a group of sets is the set of elements that belong to every set in the group. For example, the intersection of the sets, {1, 2}, {1, 3} and {1, 4} is {1}. The union of a group of sets is the set of elements that belong to any set in the group. For example, the union of the sets, {1, 2}, {1, 3}, and {1, 4} is {1, 2, 3, 4}.

Loosely speaking, two or more sets are said to be disjoint if they have no element in common, this is often stated as their intersection being empty. For example, {1, 2, 3} and {4, 5, 6} are said to be disjoint sets, whereas {1, 2, 3, 4} and {4, 5, 6} are not; in this case, one says that they “overlap.” More technically, a set of disjoint sets is a set whose members are sets that have no element in common; the set {{1, 2, 3}, {4, 5, 6}} is a set of disjoint sets.

#### Cover of S

A (non-empty) set Z of non-empty subsets of S is called a cover (or covering) of S if the union of Z’s members is the original set S. For example, there is only a single cover of {1}, namely {1}. However, there are five covers of *Y* = {1, 2}, namely:

1. {{1}, {2}},
2. {{1, 2}},
3. {{1}, {1, 2}},
4. {{2}, {1, 2}},
5. {{1}, {2}, {1, 2}}.

As this example shows, the subsets in a cover can overlap.

Examples of sets of subsets of Y that do not cover it are: {{1}} and {{2}}.

If Z is a cover of S, then Z is also a powerset-subset of S. Hence, the Cover-Of relation is a subset of the Powerset-Subset-Of relation, formally:

12 $$\begin{aligned}&{\textit{Cover-Of}} \equiv {\hbox {CO}} \end{aligned}$$

13 $$\begin{aligned}&\forall x\,\forall y [(<x,y>\in {\hbox {CO}})\rightarrow (<x,y>\in {\hbox {PSO}})] \nonumber \\&\quad \equiv ({\hbox {CO}}\subseteq {\hbox {PSO}}) \end{aligned}$$

The powerset can be divided by cover into two sets: the covering sets and the non-covering sets. Every set of subsets of S falls into one or the other of these two. Of course, one needs to know S to determine which side the set goes.

#### Partition of S

A partition of a set S is a set of disjoint subsets whose union is S, in other words disjoint subsets of S that cover S. If Z is a partition of S, then Z is also a powerset-subset of S. Hence, the Partition-Of relation is a subset of the Cover-of and Powerset-Subset-Of relations, formally:

14 $$\begin{aligned}&{\textit{Partition-Of}} \equiv {\hbox {PaO}} \end{aligned}$$

15 $$\begin{aligned}&\forall x\,\forall y\, [(<x,y>\in {\hbox {PaO}})\rightarrow (<x,y>\in {\hbox {CO}})]\nonumber \\&\quad \equiv ({\hbox {PaO}} \subseteq {\hbox {CO}}) \end{aligned}$$

16 $$\begin{aligned}&({\hbox {PaO}} \subseteq {\hbox {CO}}) \wedge ({\hbox {CO}} \subseteq {\hbox {PSO}}) \rightarrow ({\hbox {PaO}} \subseteq {\hbox {PSO}}) \end{aligned}$$

The partition-set of a set S is the set of all partitions of S. A set of size *n* (i.e., with *n* members) can be partitioned into a fixed number of non-empty subsets; in other words, the partition-set has a fixed number of members. This can be calculated and is known as the Bell number. For example, there are five ways a three-membered set can be partitioned; so the partition-set has five members. This means that the Bell number for a set of size = 3 is 5. For example, the set of numbers {1, 2, 3} can be partitioned as follows:

1. {{1}, {2}, {3}}
2. {{1, 2}, {3}}
3. {{1}, {2, 3}}
4. {{1, 3}, {2}}
5. {{1, 2, 3}}

The set of these five subsets is the set of partitions of the set {1, 2, 3}. Each one of the five members of this set is a partition of the set {1, 2, 3}. For each member partition, the union of all its members is the set {1, 2, 3}.

The Bell number increases quickly, so for size = 10 it is 115,975.

The partition-set of a set S is a subset of the powerset of S.

An incomplete partition of a set S is a collection of disjoint subsets whose union is a subset of S, but not S itself (also known as a proper subset of S), in other words disjoint subsets of S that do not cover S. For example, the set of numbers {1, 2, 3} can be incompletely partitioned as:

1. {{1}, {2}}
2. {{1}, {3}}
3. {{2}, {3}}
4. {{1, 2}}
5. {{2, 3}}
6. {{1, 3}}
7. {{1}},
8. {{2}} and
9. {{3}}.

### Reifying the operations

Conceptual modeling prefers a declarative style, where things such as the relation between a set and its powerset are treated explicitly as a relation rather than, as in logic textbooks, as an operation.

#### Powerset-of relation

The relation between a set and its powerset has already been identified as one-to-one. Also, from the above definitions, it is known that the set is a member-of the powerset—as it is a subset of itself. So for each set-powerset combination, there is a unique member-of relation that links them; these are labeled powerset-of relations. More formally,

17 $$\begin{aligned}&{\textit{Powerset-Of}} \equiv {\hbox {PO}} \end{aligned}$$

18 $$\begin{aligned}&\forall x\,\forall y\,[(<x,y>\in {\hbox {PO}})\rightarrow (y\in x)]\, {\hbox {equivalent to}} \end{aligned}$$

19 $$\begin{aligned}&\forall x\,\forall y\,[(x=\wp (y))\rightarrow (y\in x)] \end{aligned}$$

#### Powerset-subset-of

The simplest declarative solution is to deconstruct this into two already existing relations. Saying that *x* is a powerset-subset of *y* is equivalent to saying that *x* is a subset of the powerset of *y*. More formally,

20 $$\begin{aligned}&{\textit{Powerset-Subset-Of}}\,(x, y) \equiv {\hbox {PSO}} (x, y)\end{aligned}$$

21 $$\begin{aligned}&{\hbox {PSO}}\,(x,y)\equiv x \subseteq \wp (y) \end{aligned}$$

### A technical point

#### The powerset axiom and the universal set

Though this is a technical matter and only indirectly of concern here, it is worth being aware that the topic exists. One of the areas of study in set theory is the universal set, see Church [18], Barwise and Moss [9], and Forster [35]. This is the set that contains all other sets as members, it is a way to formalize the statement “*x* is a set”; this becomes “*x* is a member-of the universal set.”

However, it turns out that if one wants to include this in one’s formalization, then there are a number of formal trade-offs that need to be considered. One trade-off relates to the ZF powerset axiom which says that every set has a powerset. This is problematic as the cardinality (the number of members) of a powerset is always greater than the original set. If one adopts this axiom as it stands and the universal set, then one arrives at an inconsistency. The powerset is a set and so a member-of the universal set. Every instance of the powerset is a set, and so a member-of the universal set, hence the powerset is a subset of the universal set. But it has more members than the universal set which is impossible.

There are a number of technical ways of accommodating this. ZF avoids the problem by having no universal set. Church [18] proposed a weaker powerset axiom. Quine proposed, in New Foundations, a subset of Cantorian sets to which the cardinality of the powerset axiom applied. While it is important to have a consistent formal structure, the particular way of dealing with this issue does not affect the topic of this paper, and so is outside the scope.

#### The extensionality of set theory

Set theory is extensional. This means that the extension, the members, of the set do not change and that two sets with the same extension (members) are the same set. This is an extraordinarily powerful criterion of identity. Any formal theory of a domain will need to provide a semantics and face issues such as accounting for change over time and possible members; our use of set theory here is no different. The standard way to do this is through the use of a four-dimensional, possible world semantics, see Lewis [67], and we assume a similar semantics for our benchmark. Some of the classification systems we review later in the paper explicitly adopt this semantics.

### Alternative mathematical frameworks

Earlier it was noted that there are alternative foundational mathematical frameworks—type theory and category theory which contain objects with an analogous structure to set theoretic objects described above. These are very technical subjects, but for completeness, a very brief description is provided in this section along with references.

**Fig. 8:**
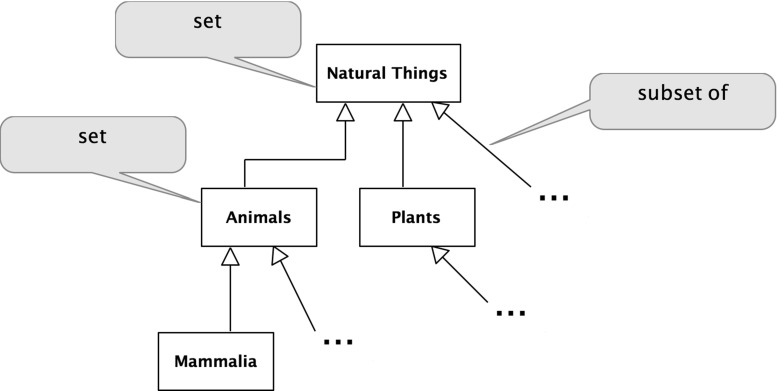
Taxonomic nodes as sets

#### Type theory

Russell [103] introduced the first type theory in 1903. Computer scientists have found a later type theory, Martin-Löf [74] type theory, useful. Mathematicians have more recently developed this into homotopy type theory [114].

What distinguishes set theory and type theory is that in set theory, objects are assumed to exist independently, whereas in type theory each object is assumed to be dependent upon its type. For example, in set theory, the set {1, 2, 3} is assumed to exist. In type theory, one might say that the set {1, 2, 3} exists and is of type SET. To illustrate the difference, in type theory everything has to have a type, so one has to ask what type the object SET is. One could say it is of type TYPE and, to stop an infinite regress, say the object TYPE is of type TYPE. Barwise and Moss [9] discuss the logical issues this circularity creates.

#### Category theory

Eilenberg and MacLane [29] introduced categories as a formal ground for what they called functors and natural transformations. Since then, they have evolved significantly. Though Grothendieck [45], Freyd [38], and others chose for practical reasons to define categories in set-theoretic terms, subsequently sets have been treated as a kind of category, a special kind of topos.

Category theory formalizes mathematical structures into categories that are collections of objects and arrows (also called morphisms) that satisfy some basic conditions. There is a category of sets, where the objects are sets and the arrows are functions from one set to another (though the objects of a category need not be sets nor the arrows functions). Any way of formalizing a mathematical concept such that it meets the basic conditions on the behavior of objects and arrows is a valid category, and all the results of category theory will apply to it.

The relationship between categories and sets is quite technical—see Blass [11] for an overview—and is outside the scope of this paper.

## Formalizing classifications using mathematical set-theoretic objects

The formal structures captured by powerset and its related mathematical objects, described in the previous section, are sufficient to characterize the core formal structure of classifications. In particular, it provides tools to examine the formal structure of the classical Linnaean system introduced earlier. This is traditionally considered taxonomical. This is true, but as the following analysis shows, the implicit pattern underlying the Linnaean system is more intricate than a mere taxonomy (i.e., hierarchy just based on subsets).

### The Linnaean taxonomy

Figure 1 above presented the explicit Linnaean taxonomy which can be interpreted formally. A natural interpretation for this, as for many taxonomies, is of the classification nodes as sets, as they have members. *Natural Things* is the set of all natural things, *Animals* is the set of all animals, and so on. From this, it naturally follows that the relationship between these sets in the taxonomic hierarchy is a subset relation. For example, *Animals* is a subset of *Natural Things*; every member-of *Animals* is also a member-of *Natural Things*. The subset schema is:

22 $$\begin{aligned} x \subseteq y\,\hbox {iff}\,\,\forall z\,(z \in x \rightarrow z \in y). \end{aligned}$$

**Fig. 9:**
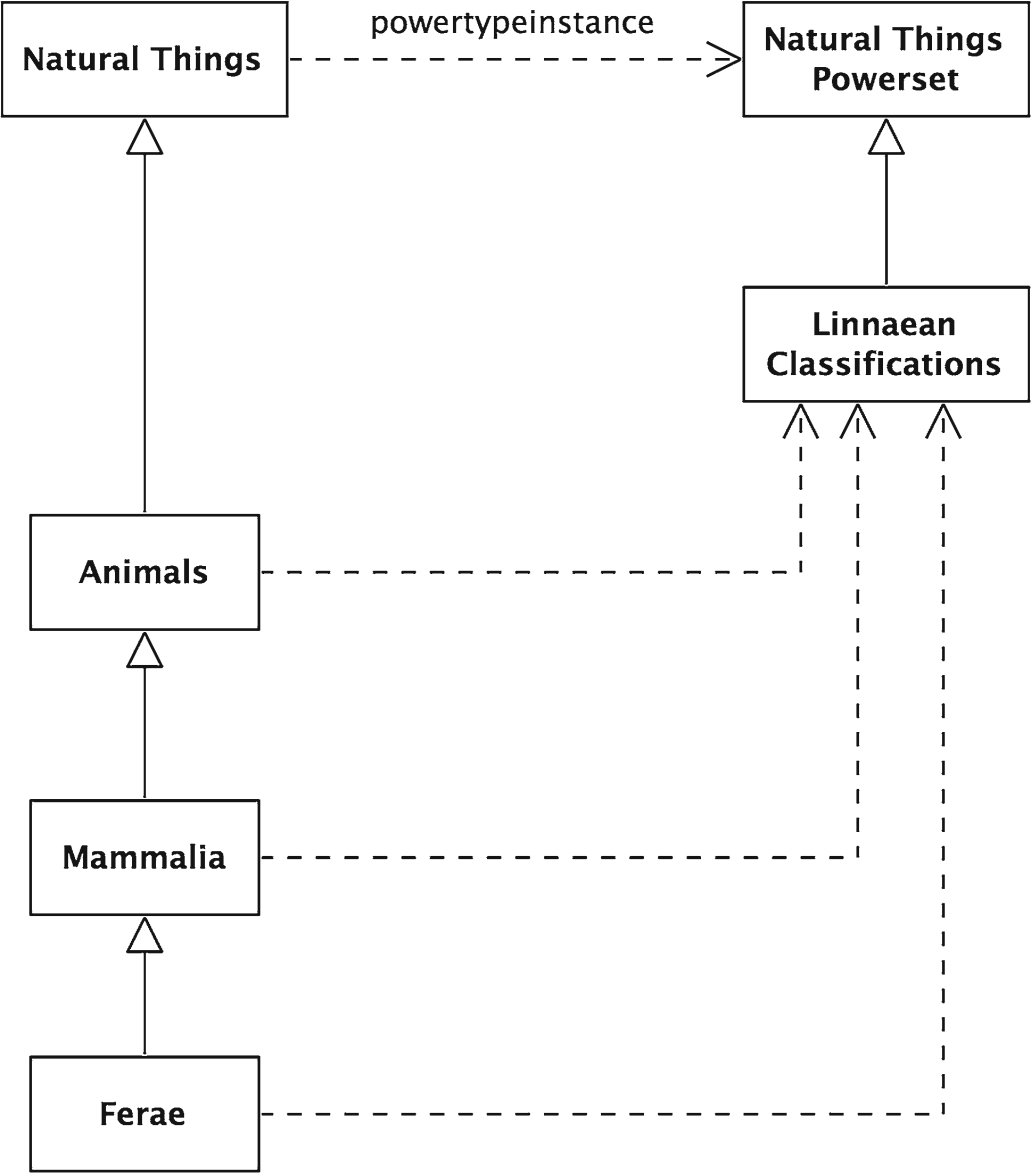
Linnaean classifications

Translating this into the current context:

23 $$\begin{aligned}&{\textit{Natural Things}} \equiv {\hbox {NT}} \end{aligned}$$

24 $$\begin{aligned}&{\textit{Animals}} \equiv {\hbox {An}} \end{aligned}$$

25 $$\begin{aligned}&\forall z\,(z \in {\hbox {An}} \rightarrow z \in {\hbox {NT}}) \end{aligned}$$

This implies that:

26 $$\begin{aligned} {\hbox {An}} \subseteq {\hbox {NT}} \end{aligned}$$

This interpretation is shown in Fig. 8.

### The Linnaean classifications

As *Natural Things* is a set, any arbitrary collection of its members is a subset. Only a select few of these are Linnaean Classifications. For example, arbitrary unions of the classifications, such as the union of Mammalia and Plants, are not. This can be made explicit by reifying the selected sets as members of the set *Linnaean Classifications*. This is a subset of the powerset of *Natural Things*, *Natural Things Powerset*.

27 $$\begin{aligned}&{\textit{Natural Things Powerset}} \equiv {\hbox {NTP}} \equiv \wp ({\hbox {NT}}) \end{aligned}$$

28 $$\begin{aligned}&{\textit{Linnaean Classifications}} \equiv {\hbox {LC}} \nonumber \\&\quad \equiv \{{\textit{Animals, Plants}}, \ldots , {\textit{Primates}}, \ldots , {\textit{Felis tigris}}, \ldots \} \nonumber \\ \end{aligned}$$

29 $$\begin{aligned}&{\hbox {LC}} \subseteq \wp ({\hbox {NT}}) \end{aligned}$$

It is assumed that the powerset relation has been reified as a powerset-of relation as described above. Then, $<$ *Natural Things Powerset, Natural Things* $>$ is an instance of the powerset-of relation; formally,

30 $$\begin{aligned} \exists x\,[(\hbox {x}\equiv <\wp ({\hbox {NT}}),{\hbox {NT}}>) \wedge (\hbox {x} \in {\hbox {PO}})] \end{aligned}$$

This is modeled in Fig. 9. The dashed line with an open arrowhead represents the member-of (i.e., type-instance) relation while the continuous line with closed arrowhead represents the subset-of relation. Since the powertype instance relation is a type of type-instance relation, a similar notation is used.

### The five Linnaean ranks

Figure 1 shows the five Linnaean ranks as levels in the taxonomy. The question is how to interpret these. A natural interpretation of their formal structure is as a set of the sets in that rank. So, for example, the rank *Orders* is the set {*Primates*, *Bruta*, *Ferae*, ...}, and so *Bruta* is a member-of *Orders*, more formally:

31 $$\begin{aligned}&{\textit{Orders}}= {\textit{Or}}=\{{\textit{Primates, Bruta, Ferae}}, {\ldots }\} \end{aligned}$$

32 $$\begin{aligned}&{\textit{Bruta}} = {\hbox {Br}} \end{aligned}$$

33 $$\begin{aligned}&{\hbox {Br}} \in {\hbox {Or}} \end{aligned}$$

As the subset relation is transitive and given the interpretation above, it follows that every Linnaean classification node is a subset of all the nodes above it in the taxonomic hierarchy. In particular, it is a subset of the root node, *Natural Things*. The full formal analysis for *Felis leo* is below.

34 $$\begin{aligned}&[({\textit{Felis leo}} \subseteq {\textit{Felis}}) \wedge ({\textit{Felis}} \subseteq {\textit{Ferae}})]\nonumber \\&\quad \rightarrow ({\textit{ Felis leo}}\subseteq {\textit{Ferae}}) \end{aligned}$$

35 $$\begin{aligned}&[({\textit{Felis leo}} \subseteq {\textit{Ferae}}) \wedge ({\textit{Ferae}} \subseteq {\textit{Mammalia}})]\nonumber \\&\quad \rightarrow ({\textit{Felis leo}} \subseteq {\textit{Mammalia}}) \end{aligned}$$

36 $$\begin{aligned}&[({\textit{Felis leo}} \subseteq {\textit{Mammalia}}) \wedge ({\textit{Mammalia}} \subseteq {\textit{Animals}})] \nonumber \\&\quad \rightarrow ({\textit{Felis leo}}\subseteq {\textit{Animals}}) \end{aligned}$$

37 $$\begin{aligned}&[({\textit{Felis leo}} \subseteq {\textit{Animals}}) \wedge ( {\textit{Animals}} \subseteq {\hbox {NT}})] \nonumber \\&\quad \rightarrow ({\textit{Felis leo}} \subseteq {\hbox {NT}}) \end{aligned}$$

**Fig. 10:**
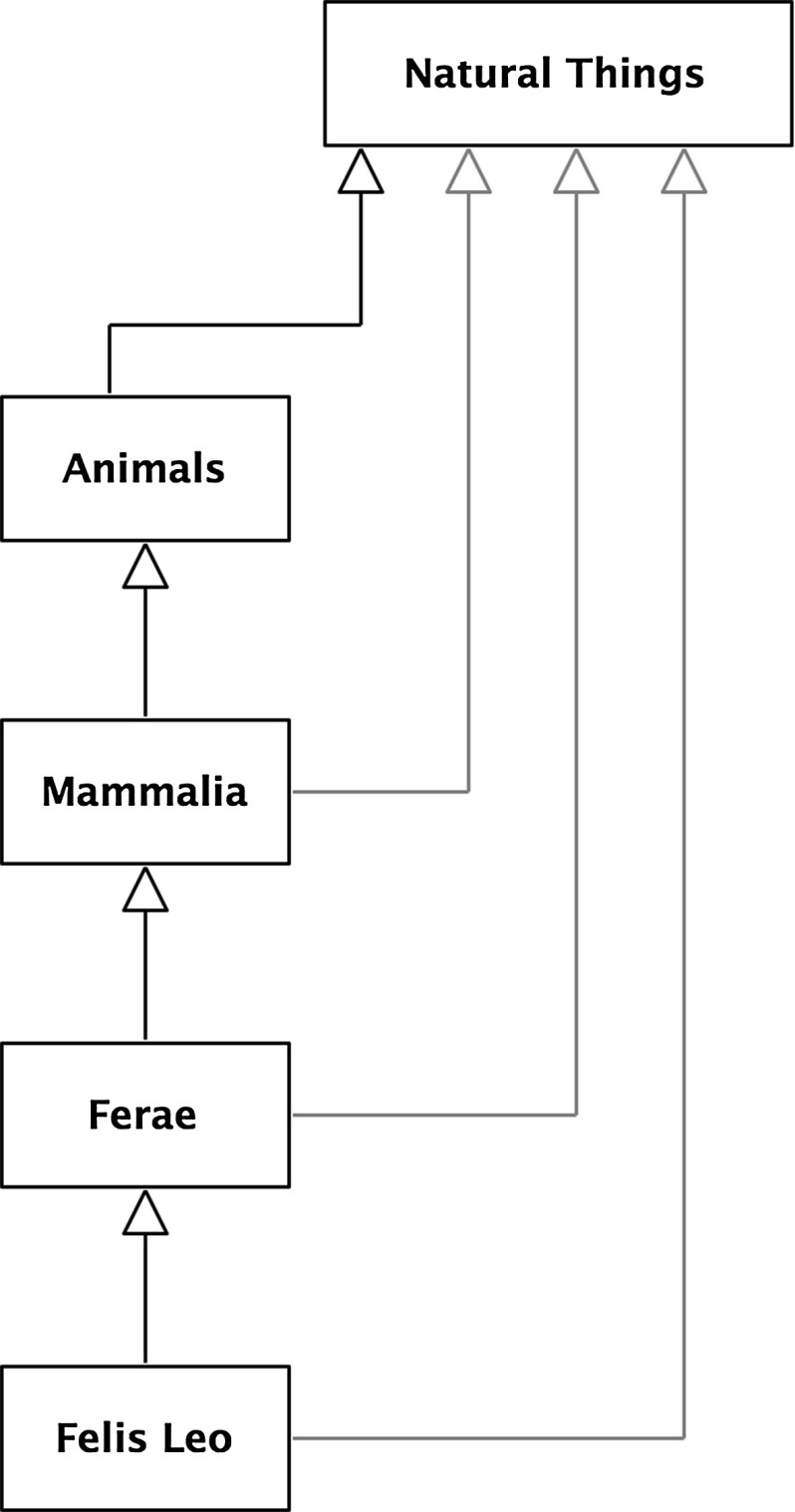
Subsets of the root node

**Fig. 11:**
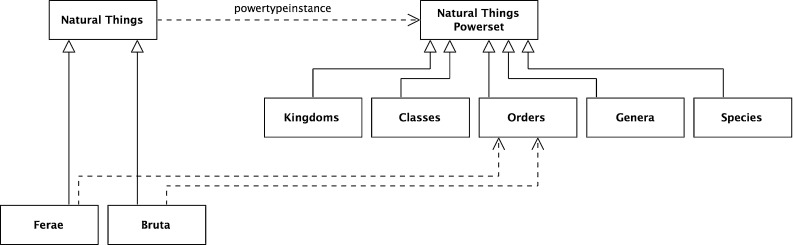
Ranks as subsets of natural things powerset

So the Species *Felis leo*, its parent *Felis*, and all the nodes above it are subsets of the root node, *Natural Things*, as shown in Fig. 10, with the new subset-of relations shaded gray—this transitivity is also shown in Fig. 5.

With these subset relations exposed, a natural extension is to see ranks as a set of subsets of the root node, *Natural Things*. A more formal way of expressing this is that each rank is a subset of the powerset of the root node, *Natural Things Powerset*; more formally (and shown graphically in Fig. 11),

38 $$\begin{aligned}&{\textit{Natural Things Powerset}} \equiv {\hbox {NTP}} \equiv \wp ({\hbox {NT}}) \end{aligned}$$

39 $$\begin{aligned}&{\textit{Orders}} \equiv {\hbox {Or}} \end{aligned}$$

40 $$\begin{aligned}&{\hbox {Or}} \subseteq \wp ({\hbox {NT}}) \end{aligned}$$

In Fig. 1, there is no explicit Linnaean ranks object. It is implicit, implied by a virtual column on the right-hand side of the figure. It can now be made explicit. It is the set of the individual Linnaean ranks; more formally,

41 $$\begin{aligned}&{\textit{Linnaean Ranks}}\equiv {\hbox {LR}}\nonumber \\&\qquad \equiv \{{\textit{Kingdoms, Classes, Orders, Genera, Species}}\} \end{aligned}$$

**Fig. 12:**
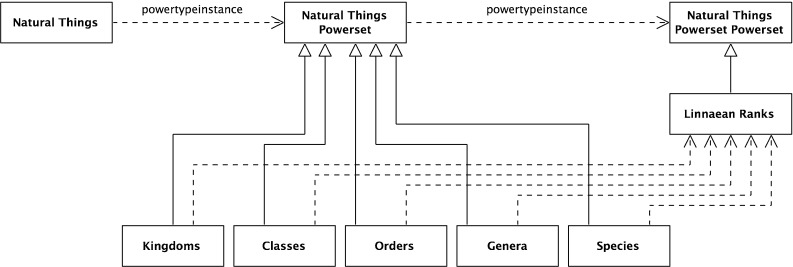
Linnaean ranks as an object

Again, the *Linnaean Ranks* can be tied back to the root, by noting that *Linnaean Ranks *is a subset of the *Natural Things Powerset Powerset*; more formally (and shown in Fig. 12 with the reified powerset-of member relation),

42 $$\begin{aligned}&{\textit{Natural Things Powerset Powerset}} \equiv {\hbox {NTPP}} \equiv \wp (\wp (\hbox {NT})) \nonumber \\ \end{aligned}$$

43 $$\begin{aligned}&{\hbox {LR}} \subseteq \wp (\wp ({\hbox {NT}})) \end{aligned}$$

*Linnaean Ranks* is a subset of *Natural Things Powerset* *Powerset* and *Linnaean Classifications* is a subset of *Natural Things Powerset*. There is a relationship between the two; members of *Linnaean Ranks* are also subsets of *Linnaean Classifications*. More formally,

44 $$\begin{aligned}&{\textit{Linnaean Ranks}} = {\hbox {LR}} \end{aligned}$$

45 $$\begin{aligned}&{\textit{Linnaean Classifications}} \equiv {\hbox {LC}} \end{aligned}$$

46 $$\begin{aligned}&\forall \hbox {y}[(\hbox {y}\in {\hbox {LR}}) \rightarrow (\hbox {y} \subseteq {\hbox {LC}})] \end{aligned}$$

**Fig. 13:**
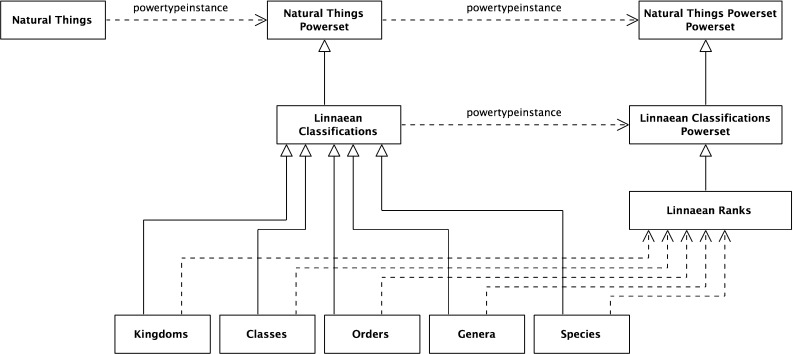
Linnaean classifications powerset

This can be expressed by using the powerset of *Linnaean Classifications* as (see Fig. 13 with the reified powerset-of relations);

47 $$\begin{aligned}&{\textit{Linnaean Classifications Powerset}}\equiv {\hbox {LCP}} \equiv \wp ({\hbox {LC}}) \end{aligned}$$

48 $$\begin{aligned}&({\hbox {LC}}\subseteq {\hbox {NTP}}) \rightarrow ({\hbox {LCP}} \subseteq {\hbox {NTPP}})] \end{aligned}$$

As the example shows, powersets are used as containers for classifications. The *Natural Things Powerset* contains *Linnaean Classifications* and the individual ranks. The *Linnaean Classifications Powerset* contains *Linnaean Ranks*. Powerset is a formal object—given the set, one can construct its powerset. There is no extra analytic or explanatory work to do; hence, it is, to use Armstrong’s [4] phrase, “an ontological free lunch.” Pragmatically, one can regard it is as a useful organizational device.

**Fig. 14:**
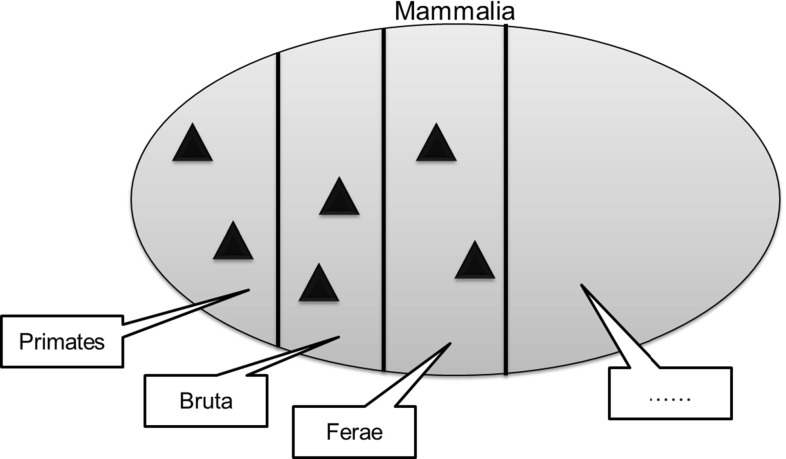
Mammalia set partitioned

### Rank ordering and partitioning

A feature of the taxonomic classification is that for each node, its subnodes at the next level partition it. For example, at the first stage, the set *Natural Things *is partitioned into the sets, *Animals*, *Plants*, etc. At the second stage, each of these sets is further partitioned; for example, the set *Animals* is partitioned into the sets *Mammalia*, *Aves*, etc. Then, the Class *Mammalia* is partitioned into *Primates*, *Bruta*, *Ferae*, and so on. Each member-of the set *Mammalia* belongs to one and only one of the subnodes (subsets)—as shown in Fig. 14.

These partitions are not explicitly specified in most classification structures. One option would be to specify each partition individually. This is less than ideal: firstly because there would be a significant number of partitions and secondly, and more importantly, because the underlying general pattern would not be specified explicitly. So the pattern would not scale well, as when new nodes were added, there would be nothing to enforce the general pattern.

**Fig. 15:**
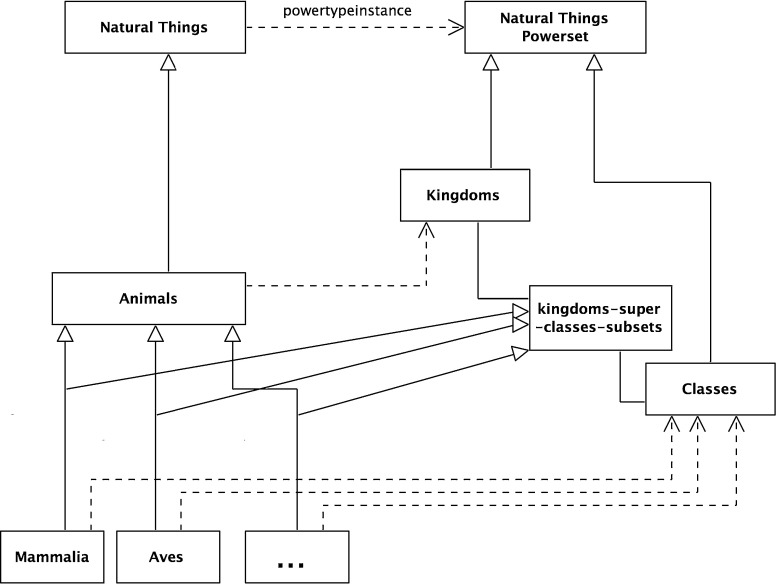
Superset-subset member pattern

A more general approach is to recognize that each rank partitions the root node, *Natural Things*, and that the ranks are ordered by the subset relation. From this, the individual partitions can be inferred.

This begins by introducing the set of partitions of *Natural Things*—*Natural Things Partitions*. *Linnaean Ranks *is a subset of this. The rank ordering is then specified by indicating that a member-of the higher node has subsets that are members of the lower node. And conversely, that a member-of the lower node is a superset of one, and only one, member-of the higher node.

**Fig. 16:**
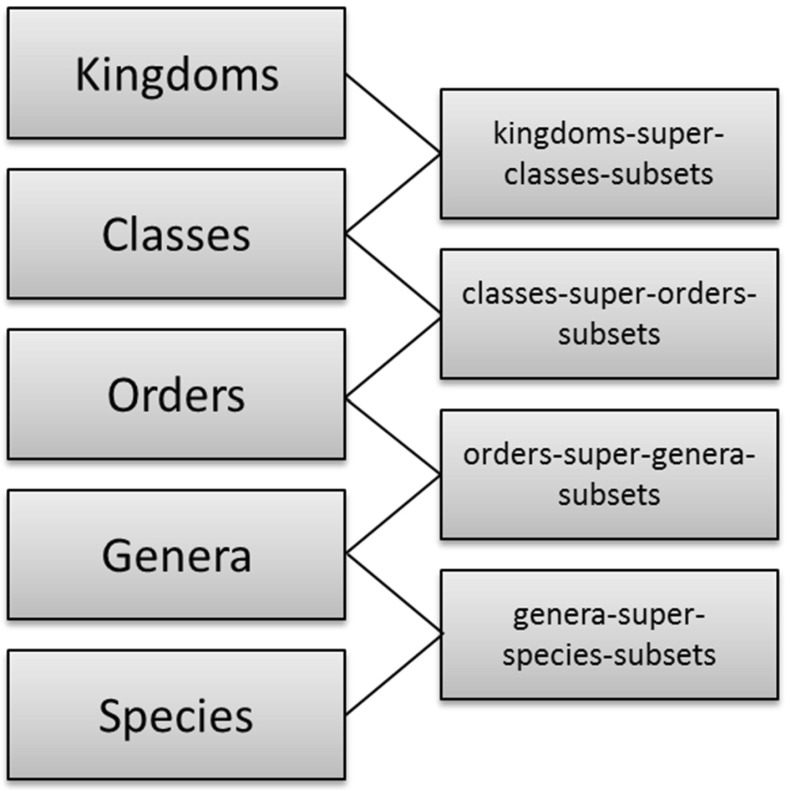
Rank ordering

For example, take Kingdoms and Classes. Every member-of Kingdoms is a superset of members of Classes, and vice versa, every member-of Classes is a subset of one and only one member of Kingdoms.

Formally, this is described as follows: recognizing the appropriate subset of the subset relation:

49 $$\begin{aligned}&K \equiv {\textit{Kingdoms}} \end{aligned}$$

50 $$\begin{aligned}&C \equiv {\textit{Classes}} \end{aligned}$$

51 $$\begin{aligned}&\forall x\,[(x \in \hbox {K}] \rightarrow \exists y\, [(y \in \hbox {C}) \wedge (y \subseteq x)] \end{aligned}$$

52 $$\begin{aligned}&\forall y\,[(y\in \hbox {C})\rightarrow \exists x \,[(x \in \hbox {K}) \wedge (y \subseteq x) \nonumber \\&\qquad \qquad \qquad \wedge \forall z\,[((z \in \hbox {K}) \wedge (y \subseteq z)) \rightarrow (x= z)]] \end{aligned}$$

From this, one can identify the class of subsets relating Kingdoms and Classes, the “kingdoms-super-classes-subsets.”

53 $$\begin{aligned}&\hbox {kingdoms-super-classes-subsets} (a, b) \equiv \hbox {kscs} (a, b) \end{aligned}$$

54 $$ \begin{aligned}&\forall x\, \forall y\,[(\hbox {kscs} (x, y)) \rightarrow ((x \in \hbox {K})\, \& \,(y \in \hbox {C})\, \& \,(y \subseteq \hbox {c})]\nonumber \\ \end{aligned}$$

It is perhaps easy to visualize this in a modeling diagram—see Fig. 15.

This pattern extends to all the ranks giving them a linear ordering—as shown in Fig. 16. Note that “kingdom-super-classes-subset” is the set of subset relations between Kingdoms and Classes. In general, there will be such a set between consecutive linear ranks. This is a good example of the usefulness of being able to build a hierarchy of subset relations.

### The underlying formal structure

The analysis has, hopefully, exposed some of the kinds of formal structures that arise in classification patterns, in particular the repeated use of powersets. The central structure is a set-subset hierarchy for selected sets. One needs to go up a powerset level to reify these selected sets into a classifications set. In the selected example, the classifications are divided into ordered ranks. One needs to go up a second powerset level to reify these ranks. The ranks are then ordered using a subset of the subset-of relation. This illustrates the ways in which the classification pattern involves a range of inter-locking formal structures generated by powersets.

The example shows how powersets can be used to characterize the formal structure of the classification pattern. One side effect of the adoption of set theory as the framework for formalization is that this leads to the introduction of types whose instances are also types—types of types. However, it is the powertypes that generate the classification pattern—types of types, by themselves are inadequate. Later, we will look at approaches that aim to characterize the classification generating pattern without the use of types of types.

## A survey of powertypes in ISE

The evolution of the formalization of classification took place in the communities working on the development of semantic or conceptual models. Their focus on how to represent domains is leading to the recognition of a requirement to represent the classification pattern formally.

However, there is another interconnecting group of communities and avenues of adoption that is of interest here. This is the general adoption of the formal mathematical structures addressed in this paper. Mathematics is an obvious tool for working with formal structures; hence, it is no surprise that communities working with computer systems adopted it. The adoption is of interest here for two reasons. Firstly, the development of conceptual modeling can be better understood when it is realized that it emerged from the early stages of a more general adoption of mathematical structures. Secondly, it provides a historical benchmark against which the less clean adoption in the conceptual modeling communities can be measured.

Broadly speaking, there is a general order of adoption of the mathematical structures. Basic notions of set and member-of in some form were adopted from the start. Subsequently, subset-of is adopted, and finally, powerset is adopted. However, the analysis shows a difference in the pace of adoption in the conceptual modeling and the main mathematics adopting ISE communities. While many mathematics adopting ISE communities absorbed the full range of objects analyzed earlier, some conceptual modeling communities have not yet completely adopted them.

In the first section below, the context is provided, describing briefly the history of the adoption of mathematical objects. In the subsequent sections, the focus is on the conceptual modeling communities. Initially, a broad outline of the adoption will be given and the main strands of development identified. Then, the various strands of development will be examined.

In the conceptual modeling communities, the literature shows clearly that the adoption of powerset was and is driven by the requirement for a classification pattern. The research shows that the adoption of powerset as part of the classification pattern is still in the process of maturing, and that the development has been in a number of different strands with differing approaches.

Unlike the mathematics adopting communities, the conceptual modeling communities have not, in general, focused on providing an account of the formal structure, though there are references to similarities with mathematical objects, such as powerset (indeed, the objects are often called powertypes). From what can be determined of the formal structure, there is a partial adoption of the mathematical objects or the development of related alternative formal structures. One of the recurring issues with some of these structures, which is described in later sections, is that they do not have the formal expressiveness of the mathematical framework detailed in this study and so often cannot support the benchmark example of this paper.

### The mathematics adopting communities

One area where the use of mathematics appeared at an early stage was the construction of database models. The first uses were focused on organizing data, rather than revealing semantics. Historically, the goal was to build database models that used data abstractions to hide the implementation details from the database user, see Smith and Smith and Smith [111], Lockemann et al. [68], Cardelli and Wegner [15], and Goldstein and Storey [40].

The database models that emerged in the early 1970s used abstractions grounded in data structures. Their primary focus was on the representation, not the represented, so they identified data objects such as records and their primary and foreign keys. They made use of mathematical objects to characterize the data object’s formal structures. For example, Codd [21, 22] introduced the relational model, which made extensive use of the notion of a set and associated set-theoretic objects, such as tuples, to capture the formal structure. Implicit in this was the use of member-of relations; subset-of relations were only used with the Cartesian product to define relations in general, and there was no evidence of powersets. In a pattern of semantic drift, this can be seen repeated elsewhere, Codd explicitly used the mathematical tuple object to develop an alternative formalization that he called “relationships”—to distinguish it from mathematical relations.

Something similar happened in the early days of structured programming, where set theoretic structures were explicitly used to characterize the data being processed. For example, Hoare [53] (p. 122) in Sect. 7 titled “THE POWERSET” (p. 122) explicitly says “The powerset of a given set is defined as the set of all subsets of that set.”

As the analysis of data structures became more sophisticated, powerset was also used by Kuper [65], Elmasri et al. [30], Gyssens and Van Gucht [46], Hull and Su [57], Soldano and Ventos [112]. Some researchers worked with type and category theory rather than set theory; Martin Löf [74] type theory was (and still is) popular, see Maietti and Valentini [70], Valentini [115], Sambin and Valentini, [104]. Cardelli [14] introduced powertypes, and these were developed by Aspinall [5]. Category theoretic approaches (such as Johnson et al. [60]) also use powersets.

By the end of the twentieth century, there were communities that had adopted the mathematical structures and used all three of the main mathematical foundational theories as a basis. From the perspective of the limited and relatively simple set of mathematical structures needed for the classification benchmark, the adoption of these was stable and mature.

### Historical context

Things are less advanced in the conceptual modeling communities. By the mid-seventies, there was a recognition that models needed to “capture more of the semantics of an application,” see Codd [23] (See also Mealy [76], Kent [62], Van Griethuysen [116], and similarly Carnap [16] originally published in 1928). The focus shifted from data toward semantics, from the representation to what was being represented. Early signs were the introduction of Abrial’s [1] semantic model and Chen’s [17] entity-relationship model.

The pattern of evolution can be seen in Chen [17], which mentions member-of—talking about an “owner-record” having a “member-record”: note the representation-oriented language. It also mentions subset-of in the text, giving an example, though there is no support for this in the notation. An extended notation was developed later by Teorey et al. [113] to support these. There was no mention of powersets.

By the 1980s, the requirements for member-of and subset-of were established, albeit under different names; Albano [2] could say “The basic abstraction mechanisms of Semantic Data Models—aggregation, classification [member] and generalization [subset]—are considered the essential features to overcome the limitations of traditional data models in terms of semantic expressiveness.” Similar sentiments can be found in a number of papers Hull and King [56], Peckham and Maryanski [96], and Hull [55]. However, powerset was the Cinderella—not mentioned.

In the 1990s, a number of papers emerged that explicitly drew attention to powersets and their use in classification—most explicitly mentioning the link to the mathematical object, though not always adopting its formal structure. There is a “road to Damascus” theme in the early literature, noting the lack of recognition of powersets in the community and their ubiquity in the domains being represented, for example, Odell [81] (pp. 23, 32) and Henderson-Sellers and Gonzalez-Perez [52].

The approach to classification is framed by an aspiration to explain as well as characterize the formal structure. It is a plausible hypothesis that this extra explanatory burden is a contributory factor to the slower adoption of the formal structures in the conceptual modeling communities.

These papers can be divided into three major strands, the first two adopting only part of the formal structures for powersets:

- Materialization-powertype
- UML-powertype (including Odell)
- BORO-powertype (including ISO 15926-2).

There are also a number of minor strands worth reviewing for completeness:

- entity-relationship (ER) type
- object-oriented (OO) type.

The following sections review the major and minor strands, describing how they address the classification pattern and the issues raised by their approach.

The section after that reviews the issues raised, building up a general picture of the current state of the art on the use of powersets to characterize the formal structure of the classification pattern.

## Major powertype strands

The three major strands emerged at roughly the same time. They are reviewed below in order of known paper submission times. However, the timing is so close that submission is not a reliable guide to order the emergence. The first strand seems to have emerged independently of the other two strands. There is a strong personal connection between the second and third strands, the primary authors from both strands acknowledge the significant influence of John Edwards [see Martin and Odell [71] (p. xiii) and Partridge [88] (p. x)].

### Materialization strand

This strand was started by two papers published in the mid-1990s. The first was Goldstein and Storey [40] and the second Pirotte [98], which references the first. Other relevant papers include some written by Goldstein and Storey [41], Dahchour et al. [26], and Pirotte and Massart [97], each typically referencing the earlier papers.

This strand has clear links with work on databases; the early papers frame the discussion in terms of data abstraction, though they also make clear they see their work in terms of semantic data modeling. In later papers, the term “abstraction” is used without the data modifier and the papers framed as conceptual modeling.

Goldstein and Storey [40] explain that the name “materialization” was chosen because the more natural name “instance-of” was “commonly used for other special relationships” noting that it is intended to “model a situation that occurs frequently in the real world and has important implications for database design.” In this and the other papers, the term “realization” is often used as an alternative to “materialization.”

Goldstein and Storey [40] examine the nature of the materialization relationship. They introduce (p. 836) two levels: conceptual and concrete. Materialization is defined as a relation between conceptual and concrete objects, for example between the conceptual “models of cars” and the concrete “individual cars.” From a mathematical perspective, these two levels are roughly isomorphic to first- and second-order sets, and the materialization relation between them is a member-of relation between the levels: first-order-set-member-of-second-order-set. However, given the framing of these links as conceptual and concrete, it is clear that a set-theoretic interpretation is not intended.

Explaining how these relate and what this relationship implies is one of the challenges the series of papers addresses. Where a materialization relation exists, some properties are shared between the levels. There are also some formal constraints (p. 837); every instance of the concrete object must materialize one and only one instance of the conceptual object and the instances of the conceptual object completely partition the instances of the concrete object. From these, it can be inferred that from a formal perspective, materialization identifies a single privileged set-theoretic partition of the second-order set into first-order sets. It is not clear how this privileged partition is identified. However, this interpretation is challenged by Fig. 2 which contain several three-level materialization hierarchies, and no indication is given which levels as conceptual and which concrete, and whether the intermediate levels are both conceptual and concrete.

Pirotte [98] move the analysis along by describing materialization hierarchies that these are partially ordered by abstractness, saying, for example, “cascades of materializations, where the more concrete class of materialization is also the more abstract class of another materialization, and so on.” Illustrating its roots in database analysis, a prime motivation is characterizing how the attributes are inherited along the materialization cascade. The constraint on the cascade is that “... it appears that a necessary and sufficient condition for materialization to be possible between two classes is that they satisfy the partial order for abstractness.” What this ordering does not provide is a formal mechanism to characterize the stratification of classes such as that provided by the intransitive member relation.

**Fig. 17:**
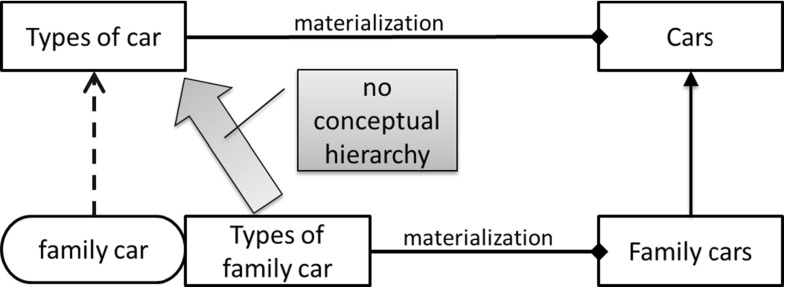
Missing conceptual hierarchy

Pirotte and Massart [97] offer a way to reinterpret the earlier conceptual approach in realist terms. In a section entitled “Real-World Modeling,” categories are introduced as the extensional counterparts of concepts, and referring to the earlier paper [98], states materialization is “a binary relationship between a class of categories and a class of more concrete objects analyzed in terms of these categories.” The paper also proposes (p. 145) a way of treating the instances of the class of categories and subclasses of the class of concrete objects as two facets of a single construct shown in the extract from its Fig. 5 below (Fig. 17). This is broadly similar to clabjects of Atkinson [6].

**Table 1:** Mapping materialization objects to their equivalent mathematical objects

Materialization object	Equivalent mathematical object
Concrete manifestation	Ur-element
Conceptual entity	First-order-set
(Types of) concrete manifestations	First-order-set
(Types of) conceptual entities	Privileged partition of a first-order set (a second-order-set)
Classification (inverse = instantiation)	Ur-element-member-of-first-order-set
Materialization	First-order-set-member-of privileged partition
Inclusion (also generalization, inverse = specialization	Subset-of
Partitioning based upon property values	Partition of S
Not recognized *	Set
Not recognized *	Member-of
Not recognized *	Subset-of
Not recognized *	Powerset

From the classification perspective of this survey, given the way in which the conceptual and concrete classes are shown, one interesting omission is a hierarchy for the conceptual objects. Figure 17 shows an extract from Fig. 5 of Pirotte and Massart [97] with a clear concrete class hierarchy on the right-hand side. The figure highlights that there is nothing directly linking the conceptual classes on the left-hand side. This is the same requirement as linking the hierarchies of ranks in the Linnaean example. For this, one needs sufficient expressivity to recognize sets of subset relations.

#### Example benchmark

The materialization papers have a narrow focus on the materialization relation rather than the wider classification pattern. Nevertheless, the materialization pattern is clearly intended to be a foundational component of the classification pattern, as the papers’ examples illustrate.

**Fig. 18:**
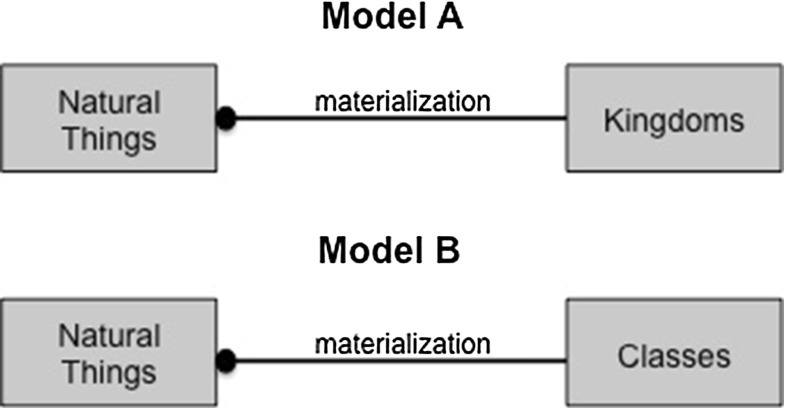
Modeling a single rank using materialization

From one perspective, these examples are similar to the Linnaean taxonomy. There is a structure where larger classes are broken down into smaller classes. There are some important differences though. Crucially, materialization is not able to represent the Linnaean ranks as ranks. For example, there is no way to stipulate that the classification has a fixed number of ranks, or indeed name those ranks or group them into a classification scheme. Finally, as noted in the last section, there is no way to show the hierarchy between the ranks. With these resources, one could represent only one rank, for example only Model A or Model B in Fig. 18, but not both.

#### Mathematical framework

The materialization papers imply that the formal structure underlying materialization is a simple partition. The materializing class of categories (the “concept”) partitions the materialized class of concrete objects into disjoint subclasses.

The materialization papers appear to assume that there is only one materializing class of categories for a class of concrete objects. A class *X* is given a materializing class typically named “Types of *X*”; for example, “Family cars” is given a materializing class “Types of family cars.” This naming convention implies that there is a single privileged materializing class; otherwise, one would expect “Type” to be qualified. But there is no explanation of why this is so, or how to pick out this privileged class from the significant number of potential partitions. The single privileged partition also makes it difficult to handle the requirement for multiple classification.

Table 1 provides a simplified mapping between the materialization objects and their equivalent mathematical objects. There is not a consistent use of terms across the materialization papers, so the first paper [40] is taken as the reference.

### Ptech-UML: Odell-powertype strand

A short time after the first materialization paper was submitted and before it was published, Odell started to put forward a view of what he named “powertype” in a series of papers [71–73, 80, 81], based upon his work at Ptech Inc. There is no indication in these that Odell was aware of the materialization work. Together, these papers give an extended and relatively informal picture of the Ptech-Odell-powertype.

Odell’s work was subsequently adopted by UML—while Odell was co-chair of the Task Force responsible for UML—and then further developed. UML can be regarded as the current owner of this strand, which here will be called Odell-UML-powertype, as the development was mostly done by Odell. However, there are important formal differences between the original Odell view and the subsequent UML development; these stages are reviewed as Odell-powertype and UML-powertype below.

At the same time as the UML development, Odell’s work on powertypes was picked up by a couple of authors Henderson-Sellers and Gonzalez-Perez [50, 51] who were dissatisfied with UML’s treatment of higher-order types. They developed an alternative view, based upon objects called “clabjects,” into which they fitted their development of the Odell account.

These three stages—Odell, UML, and clabjects—are discussed below.

#### Ptech-Odell-powertype

Odell notes [72] (p. 247) that there is an emerging recognition of this structure writing “... a particularly complex expression of categorization called power types is not addressed by traditional object structure approaches.” He subsequently notes that though previously unrecognized, it is commonplace, writing “Most systems have numerous power types, which are either unnoticed or misunderstood.”

**Fig. 19:**
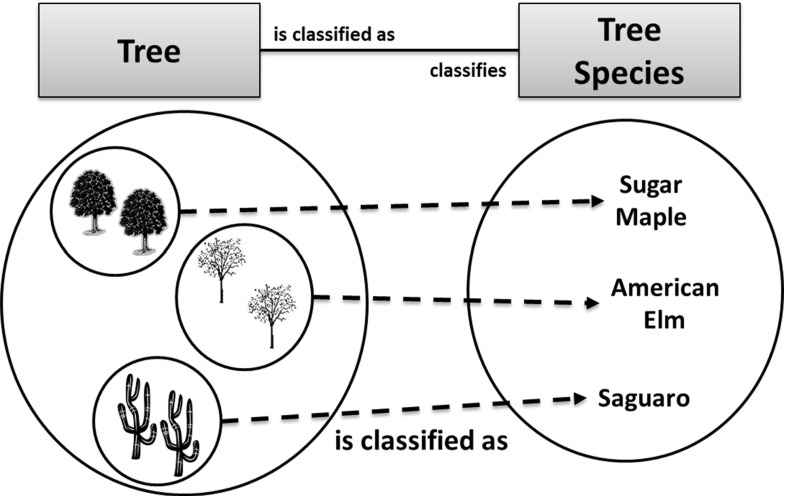
Tree species based upon [72]

In part, his approach was driven by an aspiration to explain the nature of the relationship. Hence, he characterizes his view using a prototypical example, Tree Species [72]—this is reconstructed in Fig. 19.

However, this informal approach means that some analysis is required to determine the formal structure. In Martin and Odell [71] (p. 500), there is a small section on powertype, where it is defined as all of the subtypes of the powertyped type, which looks much like the set-theoretic powerset. However, in all subsequent work, a weaker definition is used. In [72] (p. 247 and repeated in [81], p. 28), powertype is defined as follows: “A power type is an object type whose instances are subtypes of another object type.” This has marked similarities to the second half of the earlier definition of powerset “All members of $\wp$(T) are subsets of T.” Taking it at face value, this implies that an Odell-powertype is a powerset-subset.

Further analysis reveals there are two more formal constraints not mentioned in the definition that can be gleaned from the papers. Firstly, the Odell papers say that the Odell-powertype is a partition [72] (p. 255); every partition is an Odell-powertype and every Odell-powertype a partition. The equality of partition and powertype is stated without explanation. Martin and Odell [72] (p. 89) states “A type partition is a division (or partitioning) of an object type into disjoint subtypes.” It makes clear (p. 91) that “Each partition applies to all instances of an object type.” From this, it can be inferred that Odell’s partitions (and so the Odell-powertypes) are set-theoretic partitions.

Odell (p. 91) subdivides type partitions into complete and incomplete partitions based upon how they are specified in the model, defining them as follows: “A complete partition is a partition with all of its subtypes specified” and “An incomplete partition is a partition with a partial list of its subtypes specified.’ Though it is not completely clear, a reasonable inference from the use of the phrase “partial list” is that the distinction is epistemic, that is, it is about what is known rather than what exists. In other words, it is about which of the partitions subtypes are “specified” in the diagram, not whether they are in the partition. Hence, the same partition can appear in one model as complete, with all its subtypes specified and in another with only some specified. Whether it is completely or incompletely specified is about what the model “knows,” not what is actually in the partition. So this complete/incomplete distinction is immaterial from an ontological perspective.

Martin and Odell [72] (p. 254) note that a type can have multiple Odell-powertypes. It provides an example of an insurance policy that has two powertypes: one partitioned by “policy coverage type” and the other by “insurance line.” This ties in with earlier comments by Martin and Odell [72] (p. 254) that types can have multiple partitions; if Odell-powertypes are partitions and types can have multiple partitions, then partitions can, by definition, have multiple Odell-powertypes. This fits with the mathematical sense of partition, where sets can have multiple partitions—as noted earlier, in set theory this is a function of the number of elements, given by the Bell Number.

**Fig. 20:**
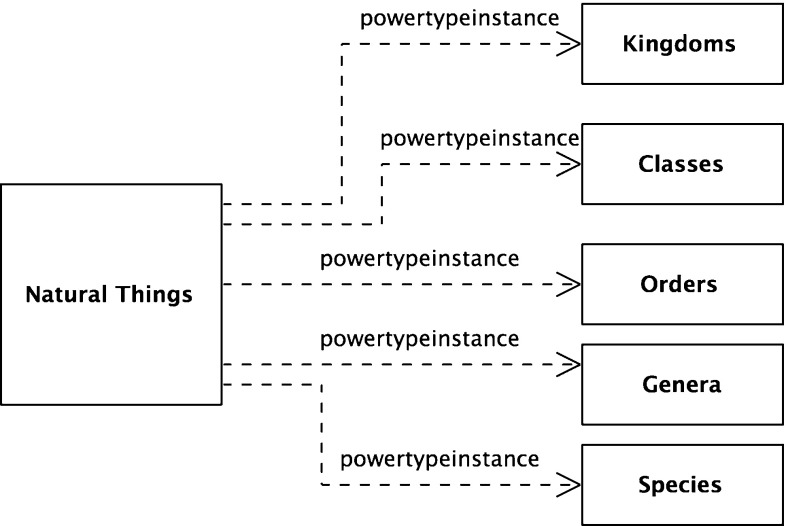
Linnaean ranks—an example of multiple Odell-powertypes

In terms of the Linnaean Classification example, the levels of classification would be multiple Odell-powertypes of the type “Natural Things”—as shown in Fig. 20—as each level is a disjoint partition at a finer level of detail.

As noted earlier, in modeling there is a preference for a declarative style where operations are reified as relations. A number of diagrams have a link between the type and the Odell-powertype, and this is labeled “is classified as.” However, there is little further characterization. A possible interpretation is the Partition-Of relation described above.

Finally, while there are no examples in the papers, there is also nothing that suggests that there cannot be higher-order powertypes—powertypes of powertypes (or subtypes of powertypes).

**Fig. 21:**
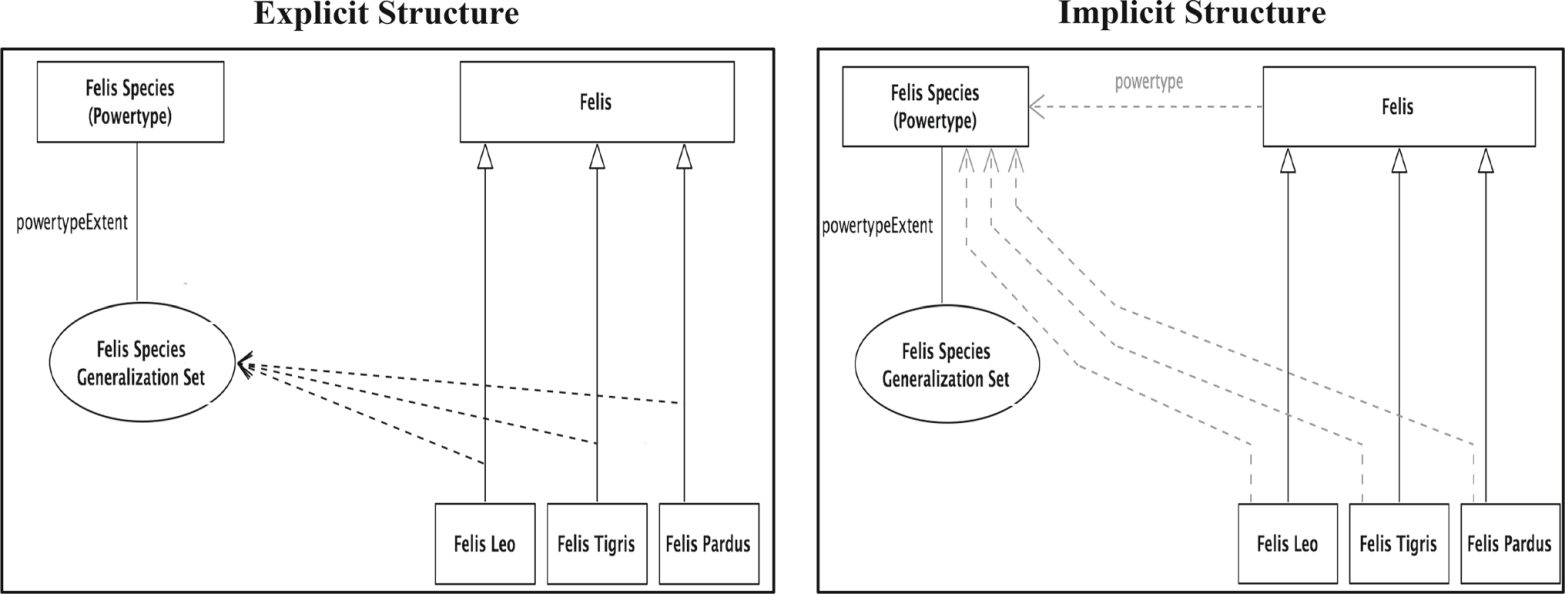
GeneralizationSet explicit and implicit structure

#### Initial mathematical framework

Comparing the Odell-Powertype with the mathematical framework definition of powerset in this paper, it is possible to note that there are a number of important differences. Firstly, the incomplete “definition” conforms to only one half of the definition of a powerset; that its instances (members) are subtypes (subsets) of the classified type. It does not conform to the other half, which would require every subtype (subset) to be a member-of the powerset. Taken by itself, this would make the Odell-powertype equivalent to powerset-subset—this, given the use of the name “powertype,” is an example of semantic drift. However, the additional constraint of the Odell-powertype being a partition of the classified type makes it a partition—a particular type of powerset-subset.

It is interesting to speculate why the full-blown power of powertype was not clearly recognized and adopted, particularly given its brief use in [71] noted earlier. A likely possible explanation is that there were not the resources to explain what a powertype is ontologically; what it is in terms of objects in the real world.

#### Comparing the Odell-powertype and the materialization strand

None of the Odell papers reference the materialization strand. However, a later materialization paper by Dahchour et al. [26] makes the connection. It refers to Odell [81] and says the Odell-powertype is the abstract class of a materialization, while the classified type is its concrete class. This accords with the analysis conducted in this survey. Dahchour et al. [26] note that the materialization refers to the relationship, whereas the Odell-powertype refers to the related object. It also notes that Odell does not use a two-faceted construction to distinguish between the instances of the powertype (the object facets) and the subtypes of the concrete class (the class facets); the merits or otherwise of separating the object and class facets are beyond the scope of this survey paper.

#### UML-powertype

As noted earlier, the “powertype” described in the evolving UML Specifications [83–85], which here is called UML-powertype, is effectively a continuation of the earlier Odell work. The specifications contain much of the same text and examples as Odell’s papers. However, its formal structure has evolved in a couple of ways relevant to this survey, giving it a stronger set-theoretic basis. These are the introduction of a GeneralizationSet, and a shifting of the original Odell distinction of complete and incomplete partitions from epistemic to ontic (where the knowledge—episteme—is incomplete rather than the partition itself) as part of a wider and finer classification of powertypes.

The specification addresses the question of what the relation between a UML-powertype and its type is through the use of a GeneralizationSet. It defines this [83] (p. 75), as “a particular set of Generalization relationships that describe the way in which a general Classifier (or superclass) may be divided using specific subtypes.” In terms of the mathematical benchmark used in this study, this is a set of subset relations—hence, a subset of the subset relation. This can be related to the UML-powertype through the use of a powertypeExtent relation. This is shown in Fig. 21, which also shows a number of other things. Firstly, it makes the GeneralizationSet explicit, using an ellipse to do this—in the UML notation, it is not explicitly shown as a separate icon, and secondly, the implied underlying relations. The relation between the type and its powertype is implied by being on the head end of all generalizations that are members of the GeneralizationSet that has a powerExtent relation with the powertype. The relation between a powertype and its instances is implied by being on the tail end of a generalization that is a member-of a GeneralizationSet that has a powerExtent relation with the powertype. As the figure illustrates, these implicit relations can be inferred—this inference is a kind or powerset-member closure referred to above.

One can see the OMG working toward formalizing this. In OMG UML 2.1 [83], this inference is explained in a tree example. In OMG UML 2.5 [85], a more formal description is given: “there is a 1-1 correspondence between instances of the powertype and specializations in the GeneralizationSet, so that the powertype instances and the corresponding Classifiers may be treated as semantically equivalent. How this semantic equivalence is implemented and how its integrity is maintained is not defined within the scope of UML.”

There is a constraint (p. 72) that each UML Generalization can only belong to one GeneralizationSet. This is described by comments such as: the set “represents an orthogonal dimension of specialization of the general Classifier.” This constraint is specific to UML-powertypes; it is not mentioned in the earlier Odell papers implying a constraint across all the UML-powertypes associated with a type, i.e., they need to be disjoint. The reason for this constraint is not explained.

The specification clarifies the historically intended relation between UML-powertype and the set-theoretic powerset saying “The notion of power type was inspired by the notion of power set.” However, it seems to have an incomplete understanding of the “notion of power set” stating incorrectly that “A power set is defined as a set whose instances are subsets.”

The second relevant change is a shift away from the original Odell distinction of complete and incomplete partitions from epistemic to ontic—using set-theoretic notions. The GeneralizationSet (*ibid.* pp. 76–77) is given Boolean attributes “isCovering,” and “isDisjoint,” where this actually classifies the related UML-powertype rather than the GeneralizationSet. The descriptions of these markings make clear that these are ontic; that they refer to the members of the powertype, not what is known about the powertype. It also makes clear through examples that these are the set-theoretic notions of cover and disjoint described earlier in the paper. This allows UML-powertypes to be of four sub-types, rather than just one partition, that is, a disjoint cover.

#### Example benchmark

The UML-powertype, like Odell-powertype, is able to meet some of the requirements for the example; for example, each of the five Linnaean ranks is a UML-powertype of the type *Natural Things*. And, like the Odell-powertype, it can meet requirements outside the scope of the example, such as multiple classification.

**Fig. 22:**
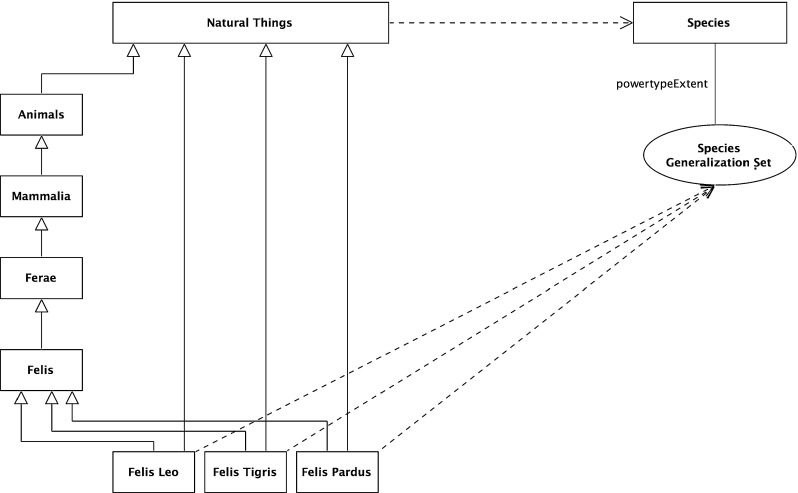
Example—proliferation of generalizations

There are some differences. The use of GeneralizationSet, where there are layers of powertypes, leads to some awkward diagramming. Each of the subtypes needs to have a Generalization link to the classified type, which in turn needs to be a member-of the powertype’s GeneralizationSet. This leads to a proliferation of Generalizations that would be unnecessary if the subtype could be directly associated with the powertype. These “extra” generalizations are shown for the Species rank in Fig. 22. This pattern is repeated for each rank.

A difficulty arises when trying to capture the relation between the ranks. This, as noted earlier, needs to work as a type-level schema linking the higher rank to the lower rank. UML does not have the resources to do this.

**Fig. 23:**
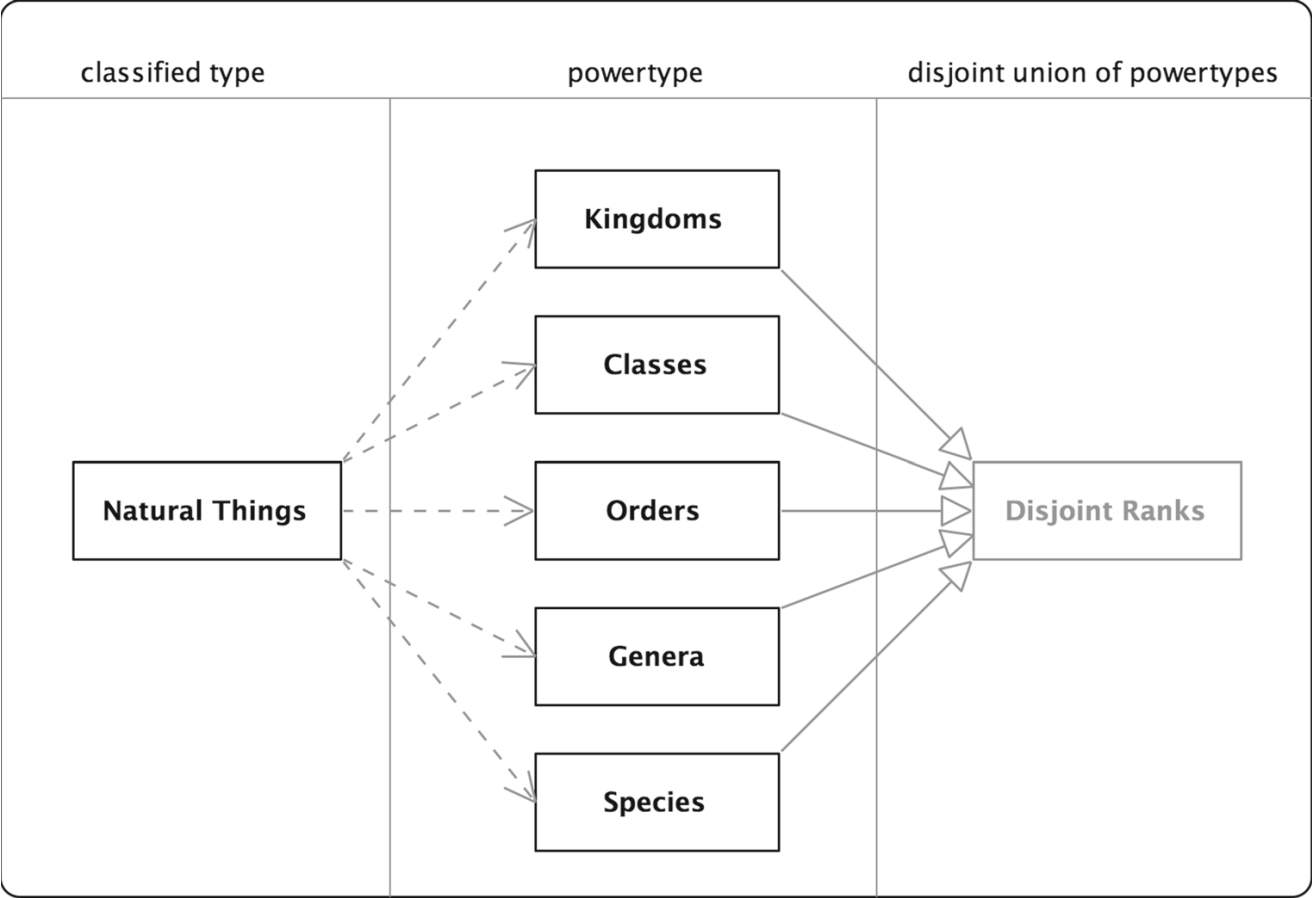
Implied GeneralizationSet constraint

There is an interesting feature of the UML-powertype range constraint. If one assumes that the Linnaean Ranks are the full set of powertypes (i.e., there are no other powertypes of Natural Things), then by the UML GeneralizationSet constraint mentioned above, the (implicit) set Linnaean Ranks is disjoint—as shown in Fig. 23.

The way the UML GeneralizationSet constraint tracks the disjoint Linnaean structure suggests a motivation for the UML constraint; it could be intended to capture the disjoint requirement in strict taxonomies such as the Linnaean classification. However, as it is currently constructed, it does not allow for either less strict classification schemes or multiple schemes (strict or otherwise) that overlap. Furthermore, where the Linnaean Ranks are not the only powertypes, there is no mechanism for UML to group the ranks into a powertype.

This is an interesting example as it illustrates the different roles that the powerset and powerset-subset patterns can play in a classification system. In both Odell and UML, there is a pair, classifying type-classified type. Linnaean Ranks look like a classifying type as its instances (ranks) are types that classify. However, to get this to work, one needs to introduce a supertype of the ranks, as the classified type. From the earlier analysis, this is known as Linnaean Classifications. While this can be introduced in UML, there is no way of constraining it to subsets of Natural Things—as UML does not have access to set-theoretic powerset.

In addition, UML has no way of representing the ordering between the ranks as it lacks access to a sufficient range of formal structures, which are characterized using set-theoretic objects in this paper. In the benchmark analysis, Natural Thing Powerset is used to do this, linking it to Natural Thing with a powertype-of relationship—see Fig. 9. It is difficult to construct the pair as UML has no candidate for the classified type; the second-level pattern is not an exact copy of the first-level pattern. One might wish to regard the Natural Thing Powerset as the classified type—but UML does not have access to set-theoretic powerset. Natural Thing Powerset has a vast number of subsets (Natural Thing Powerset Powerset has a vast number of instances), and the five Linnaean Ranks represent a very small percentage. The underlying issue is that, at this second level, Linnaean Ranks play a different organizing role to the first level. What it is organizing is a series of coarser and finer partitions of the first-level subtypes.

#### Mathematical framework

In terms of the mathematical framework, UML-powertype is a kind of powertype-subtype (a set of subsets). This is closer to the letter of the original definition and more general than the Odell-powertype. However, as noted above, there is a restriction on the range of UML-powertypes a type can have. If the set of UML-powertypes of a type is called a UML-powertype-set, then this set is a disjoint subset of the mathematical powerset of the underlying type.

As it can be seen, the formal mathematical resources that were introduced are sufficient to characterize UML-powertype-set as disjoint. As noted above, there are cases, such as the Linnaean Classifications example, where a UML-powertype-set is disjoint. However, it seems odd to make disjointness mandatory. This would suggest that the requirement is for the ability to record that some UML-powertype sets are disjoint, but not force them all to be.

Table 2 provides a simplified mapping between the Odell-UML-powertype objects and their equivalent mathematical objects.

**Table 2:** Mapping Odell-UML-powertype objects to their equivalent mathematical objects

Odell-UML-powertype object	Equivalent mathematical object
Object	Ur-element
Object type	Set
Powertype (Odell)	Partition
Is classified as (Odell)	Partition-of
Powertype (UML)	Powerset-subtype
Subtype/supertype	Subset-of/superset-of
Classify	Member-of
Partition	Partition
IsCovering (UML)	Cover-of
IsDisjoint (UML)	Disjoint set
Sub-type	Subset-of
Not recognized *	Powerset

#### Clabject-powertype

This stage can be seen as emerging from both the Odell-stage and the materialization stage. It is in this section because it explicitly builds upon the Odell approach; however, it also shares the materialization strand’s concerns about how to handle sets that are members of other sets, proposing clabjects as a solution (a topic outside the scope of this paper). Hence, this stage is named “clabject-powertype.” It is represented by a series of papers by Henderson-Sellers and Gonzalez-Perez [42, 43, 50–52] and the ISO standard 24744 [58, 59]. Its focus is on the use of powertypes for metamodeling rather than specifically on classification.

The “clabject-powertype” is called a “powertype pattern” and defined “as a pair of classes in which one of them (the powertype) partitions the other (the partitioned type) by having the instances of the former be subtypes of the latter,” see Gonzalez-Perez and Henderson-Sellers [42]. Technically, more precisely, the powertype relation is an ordered pair, as the order of the classes is material. As far as it is possible to tell, the clabject-powertype is, in set-theoretic terms, a partition. This is equivalent to the Odell-powertype, but not the UML-powertype which is not so constrained. These two are referred to directly before the definition quoted above, but the distinction between them is not noted.

The definition of Gonzalez-Perez and Henderson-Sellers [42] states that the instances of the powertype are subtypes of the powertyped type. However, an earlier paper by Henderson-Sellers and Gonzalez-Perez [51] takes a different stance, claiming that these are different facets of a single clabject (and so different), which is neither an instance nor a subtype. This is a similar position (as the paper notes) to that found in some of the materialization papers that share their concerns about how the “one over the many” (noted earlier in the discussion on sets) manifest themselves in higher-order types. This concern as noted before is outside the scope of this survey paper, and from a formal benchmarking perspective, the facets can be collapsed into a single object for comparison purposes.

As in the materialization papers, the examples (e.g., Task and TaskKind) are of a single powertype simply qualified with “...type,” implying a single preferred set-theoretic partition. There is no mention of multiple powertypes as found in the Odell papers.

#### Example benchmark

**Fig. 24:**
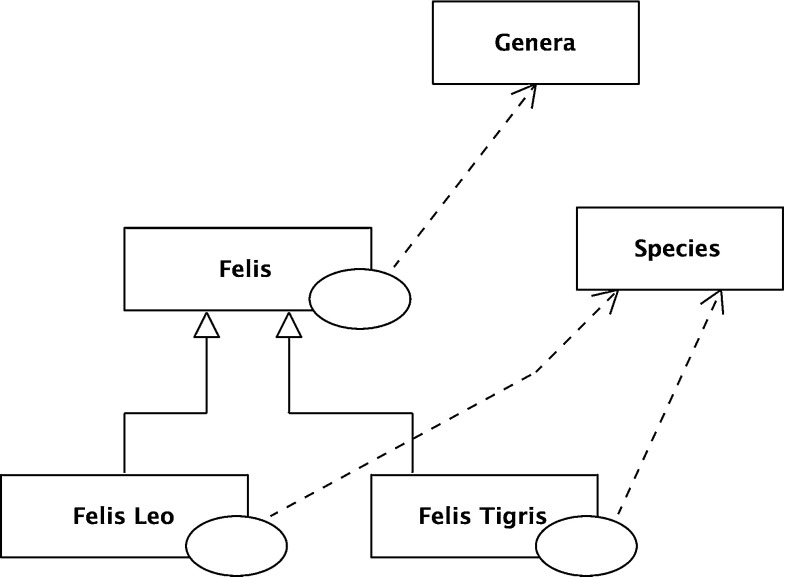
Clabject faceted ranks

It is not possible to capture the Linnaean example using this “powertype.” When only a single preferred powertype is allowed, then no more than one rank can be represented. It is not clear how the “Linnaean Ranks” can be explicitly represented with only the formal resources of set-theoretic partition. There is no mechanism for representing the relationship between the ranks. Indeed, the denial of the identity of the instance of the powertype and the sub-types of the powertyped type (in the early paper) introduce an additional layer in capturing this. Fig. 24 shows two ranks—genera and species—and the clabject facets. The classification pattern has a relationship between the two ranks that describes the sub-type relations that hold between the class-facets related to the object-facets that are instances of the ranks.

#### Mathematical framework

The clabject-powertype is a partition, and there is strong circumstantial evidence that it is, like materialization, a single preferred partition. However, again like materialization, there is no explanation of which of the many possible partitions is the preferred partition. However, see Henderson-Sellers and Gonzalez-Perez [52] where discriminants are introduced to organize partitions.

Table 3 provides a simplified mapping between the clabject-powertype objects and their equivalent mathematical objects.

**Table 3:** Mapping clabject-powertype objects to their equivalent mathematical objects

Clabject-powertype object	Equivalent mathematical object
Object (not a clabject facet)	Ur-element
Class (facet in a clabject)	Set
Instance-of	Member-of
Subtype	Subset-of
(Clabject) powertype	(Privileged?) partition
Partition	(Privileged?) partition-of
Not recognized *	Powerset-subtype
Not recognized *	Powerset

#### Semantic drift

The comparisons with the mathematical framework have made clear that the term “powertype” in the Odell-UML strand has been subject to semantic drift. It is used in this strand for variously set-theoretic partition and kinds of powerset-subset. Though materialization describes a similar type of mathematical object, as it adopts a new term, it is not a case of semantic drift.

It is not clear how aware the participants are of the semantic drift. There are clear cases of claims that the sense intended here is similar to the mathematical sense, but nowhere are the differences raised. In the case of UML, there is its mistaken definition, noted above, and the claim that it is using the mathematical sense. It should be noted that this semantic drift is confined to this strand and has not happened, for example, in the mathematics adopting communities.

This semantic drift has been noted by Halpin [48]. He says (p. 5) “If the name ‘powertype’ derives from the notion of power set (the power set of a set A is the set of all subsets of A), the term is misleading, as the powertype TreeSpecies excludes many instances in the power set of the set of trees (e.g., the null set, the set of all trees, and many other tree sets)’. For this reason, the term “higher-order type” seems more appropriate than “powertype.” The basic point is well made, but the proposed term “higher-order type” is too general as it includes incomplete partitions, so partition and powerset-subset would be more accurate.

#### Clabject’s approach to the “one over many” question

The clabjectists belong to a group within the conceptual modeling community that have a profound reluctance to accept the Cantorean resolution that objects can be both one and many. Hence, they take the view that there are no such things as types of types and have developed an alternative solution.

This appears a good example of how semantical, rather than formal, concerns have framed and so influenced the direction of the analysis. However, more analysis is required to understand the source of the semantical concerns (i.e., as noted below, the potential subject of further work). The clabject approach is framed within a multi-level modeling framework, where types are organized into levels. This framework is not suited to Cantorean sets; this is a strong motivation for looking for a different approach to powertypes.

#### A Procrustean approach?

This, in turn, could raise Procrustean concerns. In Greek mythology, Procrustes right-sized people to his iron bed, either stretching them or cutting off their feet. More analysis is required to establish whether something similar is happening here, whether the proposed solutions to the “one over many” questions are reflections of a commitment to a multi-level modeling framework.

**Fig. 25:**
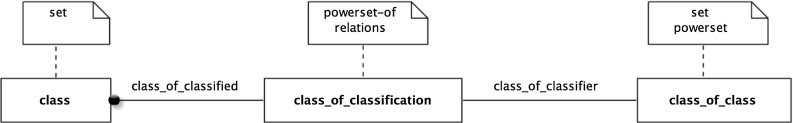
class_of_classification and class_of_class schema

### Powertype in BORO and ISO 15926-2

This strand comes from a tradition of modeling built upon a foundational, four-dimensional (4D), extensional, ontology. The use of an extensional ontology provides an explanatory context within which the mapping to extensional set theory is straightforward for all the mathematical objects under consideration. This significantly reduces the explanatory burden associated with the adoption of the full formal structure.

The core foundation was originally developed by a team of KPMG consultants working in the late 1980s and early 1990s (these projects are described by Partridge [88] (pp. xiii–xiv). In the early 1990s, the team working on EPISTLE (European Process Industries STEP Technical Liaison Executive) became aware of this work and amended their data model to accommodate 4D extensional elements. This was standardized as ISO 15926: Part 2. It has been extended in various ways, including in the work of West [118].

The results of the earlier work were documented in Partridge [88] and a series of further papers by Daga et al. [24, 25], Lycett and Partridge [69], Partridge [87, 89–92], Partridge and Stefanova [93–95] in an approach currently named BORO. More recently, this approach has been adopted by the IDEAS Group (www.IDEASGroup.org) and used to develop enterprise architecture frameworks in the USA (DODAF 2.0) and UK and Sweden (MODEM).

From this paper’s perspective, it makes sense to review ISO 15926-2 and BORO as the two main sub-strands as these illustrate the two main different formal structures within the strands.

#### ISO 15926 powertype

ISO 15926’s full title is “ISO 15926-2:2003 - Industrial automation systems and integration—Integration of life-cycle data for process plants including oil and gas production facilities—Part 2: Data model.” As the full name indicates, it was published as an ISO standard in 2003; however, the details of the model were largely finalized in the mid-1990s. The standard contains a model that is described using a standard data modeling language for product data, EXPRESS, which has been standardized in ISO 10303-11. This extends the expressivity of the model; for example, it supports keywords such as TOTAL_OVER which can be used to represent cover-of and SCHEMA declarations which can provide partitioning (partition-of).

The standard does not use the term “powerset” or any of the traditional alternatives; instead, it uses the prefix “class_of_” to indicate a powerset. At the foundation level of its hierarchy is an object “class,” which corresponds to the set-theoretic “set.” Below this in its class (set) hierarchy it has the object “class_of_class.” This is defined as “A [class_of_class] is a [class] whose members are instances of [class]”; in other words, it has as instances all subsets of “class.” From the set-theoretic perspective, this is the powerset of “class” (set). The pattern (or schema) is shown in Fig. 25. (As currently formulated, this leads to technical problems as described below.)

It also explicitly has a relation, class_of_classification, which is (in set-theoretic terms) the powerset of all member-of relations; this has as members all the individual powerset-of relations. The mapping from the rest of the ISO 15926 objects to their mathematical equivalent is relatively straightforward.

#### Mathematical framework

In terms of the mathematical framework, all its objects have equivalents as shown in the simplified mapping in Table 4.

**Table 4:** Mapping ISO15926 objects to their equivalent mathematical objects

ISO 15926 object	Equivalent mathematical object
Class	Set
Classification	Member-of
Specialization	Subset-of
class_of_ ...	... powerset
class_of_class	Set powerset
class_of_classification	Powerset-of
class_of_specialization	Member-of powerset
union_of_set_of_class	Union
intersection_of_set_of_class	Intersection

**Fig. 26:**
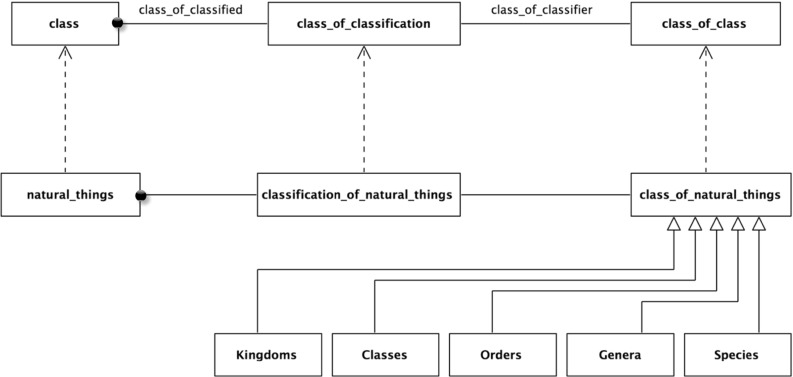
class_of_natural_things

**Fig. 27:**
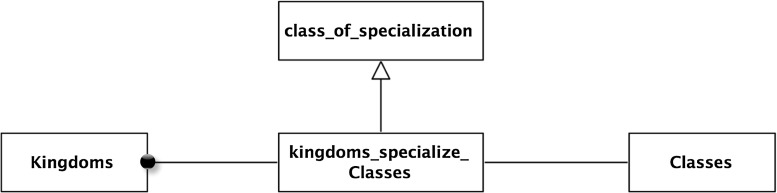
classe_of_specialization

There is however a technical issue with the way ISO 15926 formalizes the set theoretic powerset. In the standard, “class_of_class” (powerset of class) is shown as a subset-of “class” (set). This is a technical impossibility. Powersets are necessarily larger than the original set; this is the property used in Cantor’s diagonalization proof. So the “powerset of class” is necessarily larger than “class”; hence, it cannot be a subset-of (or member-of) “class.” The question of how to restrain the scope of powerset is a well-studied area of set theory with a number of technical solutions. One option adopted by ZF set theory is to avoid the universal set altogether. One of the more radical alternatives proposed by Church [18] is to have a universal set and a weakened notion of powerset, where weak powerset is the set of singletons of the original set.

#### Example benchmark

The mapping in Table 4 shows ISO 15926 has sufficient set-theoretic expressiveness to handle all aspects of the Linnaean example. Given the straightforward mapping, the details can easily be worked out from the formalization of the example given earlier. For example, the class_of_classification and class_of_class schema (Fig. 25) enables it to capture the powerset of Natural Things—shown in Fig. 26—and the powerset of the powerset of Natural Things.

It has the objects “specialization” (set-theoretic “subset-of”) and “class_of_specialization” (set-theoretic “subset-of powerset”), which enables it to capture the relations between the ranks, as shown in Fig. 27.

#### BORO powertype

The first published account of BORO (originally called REV-ENG as an acronym for REVerse ENGineering) appears in the writings of Partridge [88]. This used a terminology based upon the term “class.” During subsequent development, most lately illustrated by the International Defence Enterprise Architecture Specification for exchange (IDEAS), this has shifted to a terminology based upon the term “type.” For consistency, in this paper, the original “class” terminology shall be used.

At the foundational level of its hierarchy is an object “class,” which corresponds to the set-theoretic “set.” Powerclasses are defined (p. 307) in the usual powerset way, and the link to powerset noted: “These classes are a common feature in mathematical set theory, where they are known as powersets and defined as the set of all sub-sets of a set.” There is no restriction on the level of powerclasses; indeed, there are several examples of higher-order powerclasses. There is no semantic gap between the BORO term powerclass and the set-theoretic term powerset. Similarly, there are many examples illustrating the usefulness of multiple classification. The mapping from the BORO objects to their mathematical equivalent (in below Table 5) is even more straightforward than with ISO 15926.

#### Mathematical framework

The objects in the mathematical framework all have equivalents. Table 5 provides a simplified mapping between the BORO objects and their equivalent mathematical objects.

**Table 5:** Mapping BORO objects to their equivalent mathematical objects

BORO Object	Equivalent mathematical object
Class	Set
Class-member	Member-of
Super-sub-class	Subset-of
Power class	Powerset
Power class tuples class	Powerset-of
Intersection	Intersection-of
Fusion	Union-of
Distinct	Disjoint
Partition	Partition-of

#### Example benchmark

BORO has sufficient set-theoretic expressiveness to handle all aspects of the Linnaean example. The example is actually described in Partridge [88] (p. 206 and in Figure 9.24), though not to the same level of detail as in this paper. However, given the straightforward mapping to the set-theoretic benchmark, the rest of the details can easily be worked out from the formalization of the example given earlier.

### Implementation strands

As a number of the papers surveyed have mentioned, the classification pattern is ubiquitous. Partridge [88] provides a number of examples re-engineered from legacy systems. So it is not surprising that some form of the pattern should emerge in communities working closely with implemented systems. Two strands are briefly surveyed: one associated with entity modeling and the relational database community, and the other associated with the object-oriented community. The connections with these and earlier strands have been noted in the literature, e.g., Dahchour et al. [26].

#### Entity-relationship (ER) modeling

The entity-relationship (ER) modeling community has its roots in Chen’s entity-relationship model [17] and emerged to support the design of physical databases, typically relational databases. Within this community, there is little formal description in the literature, and it was necessary to infer the formal structure from their informal descriptions. As in other communities, this community’s understanding of the classification pattern has developed over time, and this is reflected in the literature. This is reflected particularly clearly in a series of books by Silverston [107–110]. In the initial book, there is only a brief mention of a simple pattern. In the last book in the series, a chapter is devoted to the classification pattern, in which (p. 187) the Linnaean classification is offered as a prototypical example.

In Chen [17], there was talk of classifying: “Entities are classified into different entity sets such as EMPLOYEE, PROJECT, and DEPARTMENT” (p. 11). However, there was no mention of classification of these entity sets into types, no mention of EMPLOYEE-TYPE, PROJECT-TYPE, or DEPARTMENT-TYPE. As this kind of classification became commonplace in implemented systems, it also emerged into the literature in ER modeling, for example Hay [49] (p. 44).

In this early literature, there was a simple notion of type. Although not explicitly stated, it appears to be assumed that an entity can have only one type. One indication is Hay [49] (p. 21) and Silverston [108] (p. 9) both explicitly describe naming the Type by adding the “Type” suffix to the classified entity type’s name; for example, constructing the name “Organization Type” from “Organization.” Although not stated, it is also reasonably clear that the types cannot themselves be typed—so, for example, there are no Organization Type Types. There is no explanation why these constraints exist.

Even at this early stage, it is clearly stated that the instances of the Type entities are ER-subtypes of the classified entity. However, there are constraints on what can typically be an ER-subtype, see Silverston [108] (p. 10); the “subtypes within an entity should represent a complete set of classifications (meaning that the sum of the subtypes covers the supertype in its entirety) and at the same time be mutually exclusive of each other$\ldots$”. From this, it can be inferred that not all the subsets of the entity are ER-subtypes; there is a preferred selection and they partition the supertype entity. There is no mention of how these preferred subtypes may be identified; it appears to be assumed that this is intuitively obvious.

There is some discussion of the different ways of representing subtypes, reflecting different approaches to physical implementation. Three choices are typically offered, see Hay [49] (pp. 44–45); they can be represented explicitly as subtypes, or indirectly through either a type entity or as the values of an attribute.

From a formal structure perspective, these initial simple ER types are similar to Odell-powertypes—partitioning the classified entity—with the added constraint, seen in other communities, that there is a single preferred partition.

In the later literature, there is a more sophisticated notion of classification. In Silverston and Agnew [109], three levels of classification pattern are identified ranging from simple (Level 1) to more sophisticated (Level 3). The Level 2 classification pattern allows (like the Linnaean classification) for multiple layers of classification—but (unlike the Linnaean classification) not for fixed stratified layers. It also allows for “temporary” classification, where an entity is only classified as belonging to a type for a period of time. This is done by making the classification relationship have a validity period rather than as in the BORO-type approaches classifying a stage of the entity [88] (Partridge p. xx). In a note on Silverston and Agnew [109] (p. 199), it is stated that classifications can be “mutually exclusive” or an entity can have many classifications at one time, mirroring the shift from Odell-powertypes to UML-powertypes which also introduced multiple classification. The Level 3 pattern introduced the notion of ENTITY CATEGORY TYPE, which classifies the classifications—an example would be PRODUCT CATEGORY TYPE. This corresponds with the second-order type, Linnaean classifications, in the example.

Within basic Level 3, there is a constraint on the various classification relations that any child classification can only have one parent classification. This results in a tree structure. A second Level 3—Classification Pattern with Rollups and Schemes—is introduced which allows for a many-to-many lattice structure.

While this literature does not contribute much to the formalization of the classification pattern, it is useful in showing how the understanding of the pattern has grown and is extremely useful in identifying the requirements that business systems have for the pattern. The examples for Level 3 patterns show that the Linnaean classification structure only illustrates a portion of the requirements—there is also a requirement for multiple classifications; lattice classification; multiple classification types; temporary classifications. Hence, this establishes that the Linnaean Classifications example represents a basic requirement, rather than a complete one.

#### Object-oriented patterns (minor)

Within the object-oriented (OO) community, patterns are general reusable solutions to a commonly occurring problem within a given context in software design. As such, they are often abstracted from a number of existing implementations rather than designed from scratch. Woolf and Johnson [119] describe the “The Type Object Pattern” noting that it is similar to Fowler [37], Coad [20], Hay [49], Martin and Odell [73]. The description given in Woolf and Johnson [119] and the other two papers from the OO community—Coad [20] and Fowler [37]—is at the implementation level, in terms of OO classes.

Interestingly, Woolf and Johnson [119] give an example of types of types, noting that the “Type Object pattern can be nested recursively” (see also Clark [19], Gonzalez-Perez and Henderson-Sellers [42, 43]). This indicates that they perceive no boundary on the number of levels of types.

The relationship between a type class and its (typed) class is, in the examples, one-to-many. Given the OO structures, formally one can introduce a number of type classes. However, there are no examples of multiple classifications in the paper. This suggests that the type class is formally a set-theoretic partition of the (typed) class.

## Classification formalization landscape

As the survey shows, the current treatment of classification in the conceptual modeling community is fragmented into unconnected strands. While there are some strands that have developed the full formal expressiveness required for the selected example, others have not. In this section, the landscape is characterized in terms of its range of expressiveness in two ways. Firstly, attention is given to the formal expressiveness in terms of the mathematical objects that are supported. Then, the focus turns to the inappropriate formal constraints that have emerged. Together, these give an indication of the development that is required for this area to consolidate and mature.

**Table 6:** Formal expressiveness in terms of mathematical objects

	Materialization	Odell-powertype	UML-powertype	Clabjects-powertype	BORO
Powerset					YES
Powerset-subset			YES		YES
Partition	YES	YES	YES	YES	YES
Overlapping classifications			YES		YES
Disjoint classifications			YES		YES
Cover			YES		YES
Subset subset					YES
Powerset powerset		(YES)	(YES)	(YES)	YES

**Table 7:** Formal expressiveness in terms of example requirements

	Materialization	Odell-powertype	UML-powertype	Clabjects-powertype	BORO
Multiple classification		YES	YES	(YES)	YES
Classifying subsets collection					YES
Classifications collection					YES
Classifications ordering					YES

### Classification blindness

The analysis has shown that despite the classification pattern’s ubiquity, it took a while for it to be recognized within the ISE communities. As Henderson-Sellers and Gonzalez-Perez [52] rather trenchantly observed:

“However, what is important in software engineering and modeling is that, while most people would readily discriminate between a tree and a tree species conceptually, the same cannot be said for software developers and modelers - with the exception of a small team who identified a similar notion in data modeling that they called materialization.”

As noted earlier, this can be explained, in part, by the need for “software developers and modelers” to formalize their implicit notions sufficiently and explain this formalization. Hence, it should not be surprising that the formalization did not emerge fully formed; that it needed to evolve and mature.

### Formal expressiveness in terms of mathematical objects

One thread running through the survey is the lack of sufficient formal expressiveness for the kind of classification system exemplified by the selected example. This lack is characterized in Table 6 in terms of the mathematical objects that the threads can support; YES indicates the object can be supported, (YES) indicates it seems formally possible, and a blank indicates that there is no clear indication that is can be supported.

### Formal expressiveness in terms of example requirements

The lack of expressiveness can also be characterized in terms of the example requirements. There are four main cases:

1. Multiple classifications—Linnaean rank instances
2. Classifying subsets collection—Linnaean classifications
3. Classifications collection—Linnaean ranks
4. Classifications ordering—Linnaean super-rank includes sub-rank

The main strands are mapped against these in Table 7.

### Too constraining formal structures

Finally, in some cases, the lack of expressiveness is best characterized as a too constraining structure. One thread running through the survey is the adoption of too constraining structures. There are three main cases:

1. Restricting classification to a single privileged partition.
2. Restricting classification to partitions.
3. Restricting classification to non-overlapping powerset-subsets.

The main strands are mapped against these in Table 8.

**Table 8:** Too constraining formal structures

	Materialization	Odell-powertype	UML-powertype	Clabjects-powertype	BORO
Single privileged partition	YES				
Partitions	YES	YES		YES	
Non-overlapping powerset-subsets			YES		

Some of the restrictions are more explicable than others. The restriction to partitions is sensible for some classifications. However, it is not appropriate for all as, for example, the OMG [83] makes clear. So it is good to be able to express this restriction in some case, but it should not be mandatory.

Assuming that the restriction to a partition is accepted, the further restriction to a single privileged partition is much less plausible. Maybe in some particular contexts, this kind of restriction might apply, but it does not apply in general. In addition, if one restricts oneself to a single partition, it would make sense to give some idea of what it is. Halpin [48] makes this point, taking the convention of adding a “Type” suffix as his target.

“The term “AccountType” is uninformative, because it does not provide any basis for categorizing accounts. In principle, any object type such as Account might be categorized in many different ways, leading to different types of bank account. For example, we could define an AccountKind {Local, National, International}, an AccountCategory {Taxable, Nontaxable}, and so on. These are all categorization schemes, which we may wish to use in the same model, and names such as “AccountType” and “AccountKind” don’t inform us at all about the criterion used by a given categorization scheme to place accounts into account categories.”

Kent [62] (p. 105) offers a suggestion as to why modelers are tempted down this route. He thinks it may be the experience with record-based data structures (a left over from paper technology) that is clouding the thinking:

“To fit comfortably into a record-based discipline, we are forced to model our entity types as though they did not overlap. We are required to do such things as thinking of customers and employees as always distinct entities, sometimes related by an “is the same person” relationship.”

From the perspective of this paper, the particular characteristics of individual strands are not of prime interest. What is of interest is the general picture that at the current time there is not a consistent approach to the classification pattern, nor is there an adequate formalization accepted across the community.

## Further work

Following the analysis, five areas of related further work are being considered; these are as follows:

- Large implemented systems survey
- Survey the possible semantics for powertypes
- Higher-order types survey
- Relation between higher-order types and higher-order logic
- Relation between metamodeling and higher-order types
- Ways of resolving the “one over many” question.

### Large implemented systems survey

Given the apparent ubiquity of the classification pattern, it is reasonable to assume that this pattern exists in most if not all large implemented computer systems. Hence, at the implementation level, the classification patterns must be formalized in the systems, though typically without there being a conscious appreciation that it is the classification pattern being formalized. It would be interesting to look at these formalizations to see the range of patterns and the depth of sophistication.

### Survey the possible semantics for powertypes

We assumed, for the benchmark, a standard four-dimensional, possible world semantics. We have raised the issue of the “one and the many.” However, we did not explore in detail the possible semantic and ontological issues this raised, instead focusing on formal questions. This area could be usefully explored.

### Higher-order types survey

A natural expansion of this survey would be a survey of the formalization of higher-order types in ISE. Higher-order sets are closely associated with the mathematical powerset object; in standard set theory, powersets (based upon Zermelo’s powerset axiom [120]) are typically used to generate the higher-order sets. One starts with *Ur*, the set of all ur-elements—a first-order set. The powerset of this, $\wp$(Ur), is a second-order set, with all the first-order sets as members. One can then iteratively apply the powerset operation to generate sets of any arbitrary higher order. One area that would be worth exploration is how powertypes can (ontologically) ground higher-order types (see Fine [34]).

### Relation between higher-order types and higher-order logic/language

There is also the vexed relation between higher-order types and higher-order logic to explore, in particular whether and how higher-order types require higher-order logic [48]. As [79] notes, types, including higher-order types, can, and maybe should, be included in the logical domain. In this case, first-order logic can handle higher-order types; an example of this is the standard first-order axiomatizations of set theory, such as ZF. Shapiro [106] expressed this in another way that the type order of the theory is in the semantics not the order of the language, illustrated by first-order languages that have a higher-order-type semantics. This relation between type order and logic/language could usefully be reviewed.

### Relation between meta-modeling and higher-order types

A further closely related topic is the relation between meta-modeling and higher-order types (sometimes in this context, higher-order types are called ontological meta-modeling and distinguished from linguistic meta-modeling). A survey of the formalization of this, and relating the issues to historical discussions, may help to deepen the understanding of the issues.

### Ways of resolving the “one over many” question

As noted earlier, in the ISE literature, there are a range of proposals for dealing with the “one over many” question that become more acute when powertype classification functionality is considered. This includes

- Deep instantiation, Atkinson and Kühne [7], Kühne and Schreiber [64]
- Clabjects, see Kühne and Schreibe [64], Atkinson and Kühne [8], Gonzalez-Perez and Henderson-Sellers [43]
- Multi-level objects, also known as m-objects, see Neumayr and Schrefl [77, 78].

A survey of the formalization of this and relating the issues to historical discussions on the “one over many” might help to deepen the conceptual modeling communities’ understanding of the issues. A particular area worth investigating is how far the choice of approach rests on genuine concerns relating to “one over many” or reflects commitments to other foundational assumptions. And making explicit how these various commitments relate to one another.

## Conclusions

This survey was conducted to investigate the evolution of the classification pattern in information systems engineering. The paper hypothesized that the emergence of computer systems would lead to the emergence of more sophisticated and more formalized classification patterns.

The analysis has shown that the communities within ISE have gradually adopted the formalization of the classification pattern. It has identified that the mathematical adopting communities have adopted the formal structures needed for classification more readily than the conceptual modeling communities. Analysis of the texts reveals that one of the hurdles facing the formalization in the conceptual modeling communities is providing an explanation of the formal structures needed to support classification, particularly the mathematical object powertype. This is, in part, why they lag the mathematical adopting communities and why they are currently at various different stages of formalization. Communities where there is no real explanatory burden, such as BORO, are further down the adoption route than those with more of an explanatory burden. An additional factor is the lack of sufficient formality in many of the communities.

The survey suggests there may be useful interventions. Communities can use the mathematical framework and example to identify where their structures can be enhanced. The recognition that the adoption of these formal structures needs to be supported with explanatory content will focus on its provision. These interventions in turn may lead to a mature community-wide approach to the classification pattern.

## Appendix: Tables of symbols

This paper makes use of a number of mathematical symbols and introduces a number of Linnaean classification symbols. To assist the reader, these symbols are listed in this Appendix in Tables 9 and 10.

**Table 9:** Mathematical symbols

Symbol	Name
CO (*x*, *y*)	Cover-of (*x*, *y*)
$\wp (\hbox {A})$	Powerset (A)
PaO (*x*, *y*)	Partition-of (*x*, *y*)
PO (*x*, *y*)	Powerset-of (*x*, *y*)
PSO (*x*, *y*)	Powerset-subset-of (*x*, *y*)
$x\in y$	*x* member-of *y*
$x \subseteq y$	*x* subset-of *y*
No symbol *	Intersection of S
No symbol *	Union of S

**Table 10:** Linnaean classification symbols

Symbol	Name
An	Animals
Br	Bruta
C	Classes
K	Kingdoms
kscs (*a*, *b*)	kingdoms-super-classes-subset (*a*, *b*)
LC	Linnaean classifications
LCP	Linnaean classifications powerset
LR	Linnaean ranks
NT	Natural things
NTP	Natural things powerset
NTPP	Natural things powerset powerset
Or	Orders
